# Transcriptional regulation induced by cAMP elevation in mouse Schwann cells

**DOI:** 10.1042/AN20130031

**Published:** 2014-04-30

**Authors:** Daniela Schmid, Thomas Zeis, Nicole Schaeren-Wiemers

**Affiliations:** *Neurobiology, Department of Biomedicine, University Hospital Basel, University of Basel, Hebelstrasse 20, CH-4031 Basel, Switzerland

**Keywords:** cAMP, forskolin, *in vitro*, microarray, Schwann cell differentiation, BMP, bone morphogenetic protein, cAMP, cyclic adenosine monophosphate, CNS, central nervous system, CREB, cAMP-response-element-binding protein, DAVID, Database for Annotation, Visualization and Integrated Discovery, DGC, dystrophin–glycoprotein complex, ECM, extracellular matrix, FDR, false discovery rate, GO, gene ontology, IPA, Ingenuity Pathway Analysis, Mag, myelin-associated glycoprotein, MAPK, mitogen-activated protein kinase, Mbp, myelin basic protein, Mpz/P0, myelin protein zero, NF-κB, nuclear factor κB, Olig1, oligodendrocyte transcription factor 1, PCA, principal component analysis, PFA, paraformaldehyde, PKA, protein kinase A, PNS, peripheral nervous system, qRT–PCR, quantitative RT–PCR, S.D., standard deviation

## Abstract

In peripheral nerves, Schwann cell development is regulated by a variety of signals. Some of the aspects of Schwann cell differentiation can be reproduced *in vitro* in response to forskolin, an adenylyl cyclase activator elevating intracellular cAMP levels. Herein, the effect of forskolin treatment was investigated by a comprehensive genome-wide expression study on primary mouse Schwann cell cultures. Additional to myelin-related genes, many so far unconsidered genes were ascertained to be modulated by forskolin. One of the strongest differentially regulated gene transcripts was the transcription factor Olig1 (oligodendrocyte transcription factor 1), whose mRNA expression levels were reduced in treated Schwann cells. Olig1 protein was localized in myelinating and nonmyelinating Schwann cells within the sciatic nerve as well as in primary Schwann cells, proposing it as a novel transcription factor of the Schwann cell lineage. Data analysis further revealed that a number of differentially expressed genes in forskolin-treated Schwann cells were associated with the ECM (extracellular matrix), underlining its importance during Schwann cell differentiation *in vitro*. Comparison of samples derived from postnatal sciatic nerves and from both treated and untreated Schwann cell cultures showed considerable differences in gene expression between *in vivo* and *in vitro*, allowing us to separate Schwann cell autonomous from tissue-related changes. The whole data set of the cell culture microarray study is provided to offer an interactive search tool for genes of interest.

## INTRODUCTION

Schwann cells are the glia cells of the PNS (peripheral nervous system). Throughout the entire Schwann cell lineage, both an autocrine mechanism and axon–glia interaction control the survival, proliferation and differentiation of Schwann cells (reviewed in Jessen and Mirsky, 2005). Schwann cells derive from neural crest cells, and migrate tightly associated with axons to reach distal targets. At approximately E17, Schwann cell precursors become immature Schwann cells ensheathing large axon bundles. The transition entails an orchestrated change in response to survival signals and growth factors. Around birth in rodents, immature Schwann cells differentiate into either myelinating or nonmyelinating Schwann cells. This step from an immature to a mature Schwann cell coincides with major changes in their cellular architecture. Generally, axons with a diameter larger than 1 μm are segregated to form a one-to-one relation with a Schwann cell, and thereafter will be myelinated (Peters and Muir, 1959; Voyvodic, 1989). On the other hand, small caliber axons remain engulfed by nonmyelinating Schwann cells. In addition to fiber diameter, reciprocal signaling between Schwann cells and neurons influence the Schwann cell fate; neurotrophins and growth factors, such as neuregulin1 type III were identified as regulators for Schwann cell differentiation (reviewed in Salzer, 2012). Transition into myelinating Schwann cells is also mediated by cAMP (cyclic adenosine monophosphate), which acts as a second messenger. Upon ligand binding, the intracellular heterotrimeric G protein complex activates the adenylyl cyclase, converting ATP into the second messenger cAMP (Hanoune and Defer, 2001). The PKA (protein kinase A) is activated in the presence of cAMP, which in turn stimulates the CREB (cAMP-response-element-binding protein) signal transduction pathway (reviewed in Meijer, 2009). The Gpr126 (G-protein-coupled receptor 126) is the so far only receptor identified to drive Schwann cell differentiation by elevating cAMP levels (Monk et al., 2009). Mutation in Gpr126 causes hypomyelination and retarded axonal segregation in the PNS, and cAMP elevation by forskolin treatment was sufficient to restore myelination (Monk et al., 2009; Monk et al., 2011). Elevation of intracellular cAMP has been shown to induce myelin-related gene expression such as Mpz/P0 (myelin protein zero), Krox20 (Egr2) and Galc (galactosylceramidase) in rat and human Schwann cell cultures (Lemke and Chao, 1988; Monuki et al., 1989; Parkinson et al., 2003; Monje et al., 2009). Furthermore, activation of the cAMP pathway decreases expression of proteins implicated in immature or nonmyelinating Schwann cells, such as the low-affinity neurotrophin receptor p75^NTR^, Gfap (glial fibrillary acidic protein), Gap43 (growth-associated protein 43) and cJun (Morgan et al., 1991; Monje et al., 2009). The effect of cAMP elevation was hitherto analyzed in respect to transcriptional induction of particular genes known to be important in differentiation, but its precise effect on mouse Schwann cells is not known yet.

Herein, we performed a comprehensive genome-wide expression study on primary mouse Schwann cell cultures treated with the adenylyl cyclase activator forskolin. A detailed knowledge of the effect of forskolin on Schwann cells *in vitro* is decisive, since the cAMP signaling pathway was suggested to interfere also with other signaling pathways such as the PI3-kinase and the MAP (mitogen-activated protein)-kinase pathways (Stewart et al., 1996; Kim et al., 1997; Cohen and Frame, 2001; Grimes and Jope, 2001; Ogata et al., 2004; Monje et al., 2006; Monje et al., 2010). Our comprehensive analysis identified transcriptional changes of so far disregarded genes induced by elevated cAMP levels in primary mouse Schwann cell cultures. The functional roles of most of these genes are not yet known in the Schwann cell lineage, but they might be new candidates to be considered. Furthermore, we compared the expression pattern of differentially expressed transcripts from naive and forskolin-treated cultured Schwann cells with those from sciatic nerve samples of particular postnatal developmental stages. The whole data set of the microarray study on primary mouse Schwann cell cultures is provided to offer an interactive search tool for genes of interest, analyzing their expression pattern in cultured Schwann cells upon forskolin treatment.

## MATERIAL AND METHODS

### Mice

C57BL/6 mice were kept under standard SPF-conditions, housed and treated according to the guidelines for care and use of experimental animals of the veterinary office of the Canton of Basel.

### Primary mouse Schwann cell cultures

Schwann cells were prepared from P1 (postnatal day 1) mouse sciatic nerves, and dissociated with 0.4% (w/v) collagenase and 0.125% (w/v) trypsin. DMEM (Dulbecco's modified Eagle's medium; D6546, Sigma-Aldrich) supplemented with 10% (v/v) FBS was added, and cells were seeded onto 24-well plates (Primaria™, BD Bioscience). A day after, Schwann cells were treated with 10 μM cytosine β-D-arabinofuranoside (AraC) twice for 24 h to reduce fibroblast proliferation. Schwann cells were passaged, and cells were pooled and cultured in DMEM containing 10% (v/v) FBS. For mRNA expression analysis, primary Schwann cells were seeded at a density of 25000 cells/well. For immunofluorescence analysis, 10000 Schwann cells were seeded on poly-D-lysine and laminin-coated glass coverslips in a 50 μl drop. For Schwann cell differentiation assay, cells were stimulated with 20 μM forskolin (Sigma-Aldrich) in DMEM supplemented with 10% (v/v) FBS for 24 h. Purity of mouse Schwann cell cultures determined by immunofluorescent stainings for p75^NTR^ and S100 revealed more than 85% enrichment.

### qRT–PCR expression analysis

Schwann cells were washed with PBS, and total RNA was isolated using RNeasy Micro Kit (Qiagen) according to the manufacturer's protocol. For the *in vivo* analysis, 54 sciatic nerves were pooled to nine experimental samples (*n*=9) at P0, 36 nerves were pooled to nine experimental samples (*n*=9) at P3, P9 and P21, and 16 nerves were pooled to eight experimental samples (*n*=8) for adult mice, and total RNA was isolated using ZR RNA MicroPrep™ Kit (Zymo Research). For both *in vivo* and *in vitro* studies, first strand cDNA synthesis was performed using GoScript™ Reverse Transcriptase (Promega) and random hexamer primers (Roche). Primers for qRT–PCR were designed with NCBI PrimerBLAST (Supplementary Table S1; available at http://www.asnneuro.org/an/006/an006e142add.htm). Primer pairs were chosen to overlap exon/intron junctions to prevent amplification of genomic DNA. qRT–PCR was performed on the ViiA™ 7 Real-Time PCR System (Applied Biosystems) with KAPA SYBR Fast Master Mix (Kapa Biosystems) or Power SYBR Master Mix (Applied Biosystems). The acquired mRNA copy numbers were normalized to the one of the 60S ribosomal protein subunit L13a. *In vitro* data represent the mean of 12 samples per condition derived from five independent experiments, and error bars indicate the S.D. (standard deviation). *In vivo* data represent the mean of at least eight experimental samples per time point, and error bars indicate the S.D.. Statistical quantification was performed by a Student's *t* test for unpaired groups.

### Whole-genome expression profiling

Schwann cells were stimulated with or without 20 μM forskolin for 24 h as described above. Eighteen cultures were investigated, complied by nine cultures per condition, derived from five independent experiments. The *in vivo* microarray expression analysis was performed with 28 sciatic nerves pooled to seven experimental samples (*n*=7) at P0 and P10, 20 nerves pooled to five experimental samples (*n*=5) at P4 and P7 and six nerves pooled to three experimental samples (*n*=3) at P60. Total RNA was isolated using the RNeasy Micro Kit (Qiagen) according to the manufacturer's protocol. All RNA samples had an RIN (RNA integrity number) of above 8, verified with the Agilent Bioanalyzer system (Agilent Technologies). RNA amplification, biotinylation, *in vitro* transcription and cRNA hybridization was performed as described before (Kinter et al., 2013). MouseWG-6 v2.0 Expression BeadChips from Illumina were scanned using the iScan Reader (Illumina), and global median normalization of gene expression was performed with the GenomeStudio software (version 2011.1, Illumina). One coding DNA sequence may be represented by several distinct oligonucleotides (called probes). For all examinations, probe-specific analysis was performed, allowing to identify differentially expressed transcripts with high confidence. All data passed the quality control analysis as assessed by the Illumina on-board software (GenomeStudio, version 2011.1) and by PCA (principal component analysis; Partek Genomics Suite, version 6.6, Partek Inc.). Statistical analysis was performed using Partek Genomic Suite software (version 6.6, Partek Inc.). Differentially expressed transcripts were identified by a two-way ANOVA, and *P*-values were adjusted using the FDR (false discovery rate) method to correct for multiple comparisons (Benjamini and Hochberg, 1995). Significantly differentially expressed genes were further analyzed with the IPA (Ingenuity Pathway Analysis) software (Ingenuity Systems), the DAVID (Database for Annotation, Visualization and Integrated Discovery (version 6.7) Bioinformatics Resources (Huang da et al., 2009) and TransFind (Kielbasa et al., 2010). We provided the generated database as an interactive search tool to analyze the expression pattern of genes of interest upon forskolin treatment.

### Antibodies

The following primary antibodies were used: anti-MBP (rat, 1:800, Chemicon), anti-neurofilament (mouse, 1:800, SMI31, Covance), anti-Olig1 (rabbit, 1:1000, Abcam), anti-p75^NTR^ (rabbit, 1:500, Promega), anti-S100 (rabbit, 1:500, Dako).

The following secondary antibodies were used: donkey-anti-rabbit AlexaFluor488, donkey-anti-mouse DyLight549, donkey-anti-rat AlexaFluor647 (all 1:500, Jackson ImmunoResearch Laboratories), DAPI (4′,6-diamidino-2-phenylindole) (1.25 μg/ml; Molecular Probes) was used as cellular counter stain.

### Immunofluorescent microscopy

10 μm sections of fresh frozen torso of P7 mice were mounted on gelatin/chrome alum-coated slides, dried at room temperature, and fixed for 15 min in 4% (w/v) PFA (paraformaldehyde) in PBS. Sections were washed three times for 15 min in PBS, and unspecific binding sites were impeded by incubation with blocking buffer containing 1% (v/v) normal donkey serum (Chemicon Int.), 2% (v/v) cold fish skin gelatin (Sigma-Aldrich), 0.15% (v/v) Triton X-100 (Sigma-Aldrich) in PBS for 1 h at room temperature. Primary antibodies were incubated in blocking buffer at 4°C overnight. Fluorochrome-conjugated secondary antibodies were diluted in blocking buffer, and incubated for 1 h at room temperature. Stained sections were embedded in FluorSave (Calbiochem). For stainings of Schwann cell cultures, cells were rinsed with PBS, and fixed with 4% (v/v) PFA in PBS for 15 min. Further procedure was performed as described above. Fluorescence microscopy images were acquired with the confocal microscope Nikon A1R (40× objective, numerical aperture 1.3) or Zeiss LSM 710 (63× objective, numerical aperture 1.4), using photomultiplier tube detectors. Image quantification was performed with Imaris software (version 7.6.4, Bitplane) and processing with ImageJ 1.47b software and Adobe Photoshop software (version CS5.1).

## RESULTS

### Forskolin induced transcriptional regulation of genes involved in Schwann cell development

One key signaling pathway for Schwann cell differentiation and peripheral myelination is mediated by cAMP levels. This signal transduction pathway can be activated *in vitro* by forskolin, an adenylyl cyclase activator. Since primary rat and mouse Schwann cells in cultures react distinct upon particular stimulation reagents (Yamada et al., 1995), we examined in detail the effect of forskolin on gene transcriptional regulation in primary mouse Schwann cell cultures. Schwann cells isolated from sciatic nerves of P1 mice were cultured in the presence or absence of forskolin for 24 h. We ascertained the optimal forskolin concentration of 20 μM, which resulted in robustly induced transcription of Mpz/P0, a commonly used marker for Schwann cell differentiation (D. Schmid, T. Zeis, M. Sobrio and N. Schaeren-Wiemers, unpublished work). A whole-genome expression assay was performed to identify transcriptional changes induced by forskolin treatment in mouse Schwann cells *in vitro*, and about 22000 transcripts were consistently expressed in cultured Schwann cells. The generated database is provided as an interactive search tool to analyze the expression pattern of genes of interest upon forskolin treatment (Interactive Excel file; available at http://www.asnneuro.org/an/006/an006e142add.htm). First, forskolin-dependent transcriptional expression of genes that are implicated in Schwann cell development, differentiation and myelination were analyzed (Table 1). For illustration, selected genes were schematically grouped according to their temporal expression in the Schwann cell lineage (Supplementary Figure S1; available at http://www.asnneuro.org/an/006/an006e142add.htm).

**Table 1:** Differential gene expression analysis on transcripts known in Schwann cells The expression levels of gene transcripts, which are important for Schwann cell development, differentiation and myelination were analyzed. The strongest induced mRNA expression levels were detected for Pou3f1, Egr3 and Mpz. Data are based on a two-way ANOVA, and unadjusted *P*-values < 0.01 were accounted as significant. n.s.: not significant; *: note that Mbp variants 7 and 8 are coding for Golli-Mbp, and are not expressed in myelin.

(A) Transcription factors			
Common name	Entrez ID	Ratio 20 to 0 μM	*P*-value
**Early Growth Response 1 (Krox24)**	**Egr1**	**1.68**	**0.0063**
Early Growth Response 2 (Krox20)	Egr2	1.27	n.s.
**Early Growth Response 3**	**Egr3**	**7.26**	**< 0.0001**
**Inhibitor of DNA binding 2**	**Id2**	**2.12**	**< 0.0001**
**Inhibitor of DNA binding 2**	**Id2**	**1.37**	**< 0.0001**
**Inhibitor of DNA binding 4**	**Id4**	**1.98**	**0.0118**
**Jun Oncogen (cJun)**	**Jun**	**0.62**	**< 0.0001**
Nab1, EGR-1-binding protein 1	Nab1	0.99	n.s.
Nab1, EGR-1-binding protein 1	Nab1	1.05	n.s.
Nab1, EGR-1-binding protein 1	Nab1	0.98	n.s.
Nab1, EGR-1-binding protein 1	Nab1	1.11	n.s.
**Nab2, EGR-1-binding protein 2**	**Nab2**	**1.36**	**< 0.0001**
Paired Box Gene 3	Pax3	Not detected	
**POU Domain, class 3, TF 1 (Oct6, SCIP)**	**Pou3f1**	**4.38**	**0.0181**
SRY-box Containing Gene 10	Sox10	1.05	n.s.
SRY-box Containing Gene 2	Sox2	0.88	n.s.
SRY-box Containing Gene 2	Sox2	0.93	n.s.
**AP2α**	**Tcfap2a**	**0.75**	**0.0133**
**AP2α**	**Tcfap2a**	**0.82**	**0.0139**
Yin Yang 1	Yy1	Not detected	
(B) Receptors			
Common name	Entrez ID	Ratio 20 to 0 μM	*P*-value
**v-Erb-b2 Erythroblastic Leukemia Viral Oncogene 2**	**Erbb2**	**1.34**	**0.0073**
v-Erb-b2 Erythroblastic Leukemia Viral Oncogene 3	Erbb3	Not detected	
G protein-coupled receptor 126	Gpr126	1.15	n.s.
**Nerve Growth Factor Receptor (p75^NTR^)**	**Ngfr**	**0.89**	**0.0123**
Nerve Growth Factor Receptor (p75^NTR^)	Ngfr	0.92	n.s.
Nerve Growth Factor Receptor (p75^NTR^)	Ngfr	0.92	n.s.
**Neurotrophic Tyrosine Kinase, Receptor 2 (TrkB)**	**Ntrk2**	**1.26**	**0.0045**
**Neurotrophic Tyrosine Kinase, Receptor 3 (TrkC)**	**Ntrk3**	**1.29**	**0.0018**
**Neurotrophic Tyrosine Kinase, Receptor 3 (TrkC)**	**Ntrk3**	**1.26**	**0.0068**
(C) Myelin			
Common name	Entrez ID	Ratio 20 to 0 μM	*P*-value
2′,3′-cyclic nucleotide 3′ phosphodiesterase	CNP	1.02	n.s.
2′,3′-cyclic nucleotide 3′ phosphodiesterase	CNP	1.18	n.s.
**Gap Junction Protein α1 (Connexin 43)**	**Gja1**	**1.80**	**0.0023**
Gap Junction Protein α4 (Connexin 37)	Gja4	Not detected	
Gap Junction Protein β1 (Connexin 32)	Gjb1	Not detected	
Gap Junction Protein β2 (Connexin 26)	Gjb2	1.33	n.s.
Gap Junction Protein γ3 (Connexin 29)	Gjc3	Not on the array	
**Lipin 1**	**Lpin1**	**1.37**	**0.0008**
**Lipin 1**	**Lpin1**	**1.32**	**0.0004**
Myelin-Associated Glycoprotein	Mag	0.98	n.s.
Myelin and Lymphocyte Protein	Mal	1.06	n.s.
Myelin and Lymphocyte Protein	Mal	1.37	n.s.
**Myelin Basic Protein (variant 1-7)**	**Mbp**	**0.10**	**< 0.0001**
**Myelin Basic Protein (variant 1, 2, 4)**	**Mbp**	**0.80**	**< 0.0001**
Myelin Basic Protein (variant 8)	Mbp	0.92	n.s.
**Myelin Basic Protein (variant 1-8)**	**Mbp**	**0.40**	**< 0.0001**
**Myelin Protein Zero (P0)**	**Mpz**	**3.41**	**0.0001**
Myelin Protein Zero (P0)	Mpz	1.12	n.s.
Neurofascin	Nfasc	Not detected	
**Plasmolipin**	**Pllp**	**0.64**	**0.0018**
Peripheral Myelin Protein 2	Pmp2	0.98	n.s.
Peripheral Myelin Protein 22	Pmp22	1.51	n.s.
**Peripheral Myelin Protein 22**	**Pmp22**	**1.52**	**0.0037**
Periaxin	Prx	Not detected	
(D) Lipid biosynthesis			
Common name	Entrez ID	Ratio 20 to 0 μM	*P*-value
ATP-binding cassette transporter D1	Abcd1	1.02	n.s.
ATP citrate lyase	Acly	1.10	n.s.
Aldehyde dehydrogenase family 3, subfamily A2	Aldh3a2	1.08	n.s.
Aldehyde dehydrogenase family 3, subfamily A2	Aldh3a2	1.09	n.s.
Arylsulfatase A (ASA)	Arsa	1.10	n.s.
Sterol 27-hydroxylase	Cyp27a1	Not detected	
7-dehydrocholesterol reductase	Dhcr7	1.04	n.s.
Fatty acid 2-hydroxylase	Fa2h	Not detected	
Fatty acid binding protein 7 (Blbp, Bfabp)	Fabp7	Not detected	
Fatty acid synthase	Fasn	Not detected	
Galactose-3-O-sulfotransferase 1 (Cst, Gcst)	Gal3st1	1.30	n.s.
Galactosylceramidase	Galc	1.04	n.s.
Glyceronephosphate O-acyltransferase (Dhapat)	Gnpat	0.99	n.s.
3-hydroxy-3-methylglutaryl-Coenzyme A reductase	Hmgcr	0.95	n.s.
Phytanoyl-CoA hydroxylase	Phyh	Not detected	
Sphingosine-1-phosphate receptor 1 (S1p)	S1pr1	Not detected	
SREBP cleavage activating protein	Scap	1.15	n.s.
**Stearoyl-Coenzyme A desaturase 1 (Scd)**	**Scd1**	**1.61**	**< 0.0001**
Sphingomyelin phosphodiesterase 1	Smpd1	0.95	n.s.
Sphingomyelin phosphodiesterase 1	Smpd1	1.20	n.s.
Sphingomyelin phosphodiesterase 1	Smpd1	1.20	n.s.
Sterol regulatory element binding transcription factor 1	Srebf1	1.12	n.s.
Sterol regulatory element binding factor 2 (SREBP-2)	Srebf2	1.03	n.s.
Sterol regulatory element binding factor 2 (SREBP-2)	Srebf2	1.01	n.s.
**UDP-glucose ceramide glucosyltransferase (Gcs)**	**Ugcg**	**1.18**	**0.0078**
**UDP-glucose ceramide glucosyltransferase (Gcs)**	**Ugcg**	**1.22**	**0.0172**
**UDP galactosyltransferase 8A (Cgt, mCerGT)**	**Ugt8a**	**1.16**	**0.0126**
(E) Varia			
Common name	Entrez ID	Ratio 20 to 0 μM	*P*-value
**Cadherin19**	**Cdh19**	**0.59**	**0.0003**
**Cadherin19**	**Cdh19**	**0.62**	**0.0021**
**Cadherin2 (Ncad)**	**Cdh2**	**0.80**	**0.0010**
Disks Large Homolog 1	Dlg1	Not detected	
Dedicator of Cytokinesis Protein 7	Dock7	0.99	n.s.
Dedicator of Cytokinesis Protein 7	Dock7	1.12	n.s.
Dystrophin-related Protein 2	Drp2	1.08	n.s.
Endothelin	Edn1	0.44	n.s.
**Growth Associated Protein 43**	**Gap43**	**0.50**	**< 0.0001**
Glial Fibrillary Acidic Protein	Gfap	1.10	n.s.
**Glial Fibrillary Acidic Protein**	**Gfap**	**1.52**	**0.0041**
Histone deacetylase	Hdac1	Not on the array	
Histone deacetylase	Hdac2	0.93	n.s.
**Leucine-rich repeat LGI family, Member 4**	**Lgi4**	**1.26**	**0.0105**
**Leucine-rich repeat LGI family, Member 4**	**Lgi4**	**1.87**	**0.0002**
**Neural cell adhesion molecule 1 (CD56)**	**Ncam1**	**1.39**	**0.0010**
**Neural cell adhesion molecule 1 (CD56)**	**Ncam1**	**2.20**	**< 0.0001**
**Neural cell adhesion molecule 1 (CD56)**	**Ncam1**	**1.52**	**0.0026**
Membrane protein, palmitoylated 5 (Pals1)	Mpp5	0.95	n.s.
Partitioning Defective 3 Homolog (Par3)	Pard3	1.02	n.s.
Partitioning Defective 3 Homolog (Par3)	Pard3	1.03	n.s.
Partitioning Defective 3 Homolog (Par3)	Pard3	1.04	n.s.
Phosphatase and Tensin Homolog	Pten	0.99	n.s.
Phosphatase and Tensin Homolog	Pten	1.12	n.s.
RAS-related C3 Botulinum Substrate 1	Rac1	0.91	n.s.
Ras Homolog Family Member A	Rhoa	Not detected	
Ras Homolog Family Member B	Rhob	1.05	n.s.
S100 β	S100b	Not detected	

Analysis of transcription factors revealed a strong induction upon forskolin treatment for the mRNA expression levels of Egr3 and Oct6 (Pou3f1), a major target of cAMP signaling in Schwann cells (Monuki et al., 1989) (Table 1A). Increased transcription was also detected of Krox24 (Egr1), the Egr1-binding protein 2 (Nab2) and the inhibitor of DNA binding 2 and 4 (Id2, Id4). Reduced mRNA expression levels were present for the transcription factors AP2α (Tcfap2a) and cJun (Jun), which is in accordance to their down-regulation during development *in vivo* (reviewed in Jessen and Mirsky, 2005). For Krox20 (Egr2) and Sox10, which was strongly expressed in primary Schwann cells, no significant forskolin-dependent regulation was observed. Investigation of receptors, which were implicated in Schwann cell signaling, revealed that the expression levels of the tyrosine kinase receptors ErbB2, TrkB (Ntrk2) and TrkC (Ntrk3) were significantly increased by forskolin (Table 1B). Forskolin treatment led to a small reduction of the neurotrophin receptor p75^NTR^ (Ngfr), in accordance to previous reports on rat Schwann cell cultures (Morgan et al., 1991; Monje et al., 2009). It resulted also in increased transcription of the myelin-related genes Mpz, peripheral myelin protein 22 (Pmp22) and lipin1 (Lpn1) (Table 1C). No transcriptional regulation could be detected for 2′,3′-cyclic nucleotide 3′ phosphodiesterase (Cnp), the myelin-associated glycoprotein (Mag) and the myelin and lymphocyte protein (Mal), and reduced expression levels of plasmolipin (Pllp) and myelin basic protein (Mbp) were detected in treated Schwann cells. However, sequence analysis of the Mbp probes revealed that they code also for Golli Mbp variants, having a distinct expression pattern and function during glia development compared with classical MBP isoforms (Campagnoni et al., 1993; Pribyl et al., 1996).

During myelination, the synthesis of large amounts of lipids is important for accurate myelin formation. For this reason, the effect of forskolin treatment was investigated on the regulation of genes involved in lipid biosynthesis in Schwann cells (Table 1D). Significantly increased transcription levels were detected for the stearoyl-coenzyme A desaturase 1 (Scd1), the UDP-glucose ceramide glucosyltransferase (Ugcg, Gcs) and the UDP galactosyltransferase 8A (Ugt8a, Cgt, mCerGT), the rate-limiting enzyme of the cerebroside biosynthesis (Morell and Radin, 1969).

Our data analysis revealed that forskolin treatment of cultured mouse Schwann cells led to up-regulation of a number of transcripts which are important during Schwann cell differentiation *in vivo*, and to reduced transcription of genes known to be expressed in neural crest cells and Schwann cell precursors *in vivo* (reviewed in Jessen and Mirsky, 2005).

### Forskolin-induced transcriptional regulation in Schwann cells

**Figure 1:**
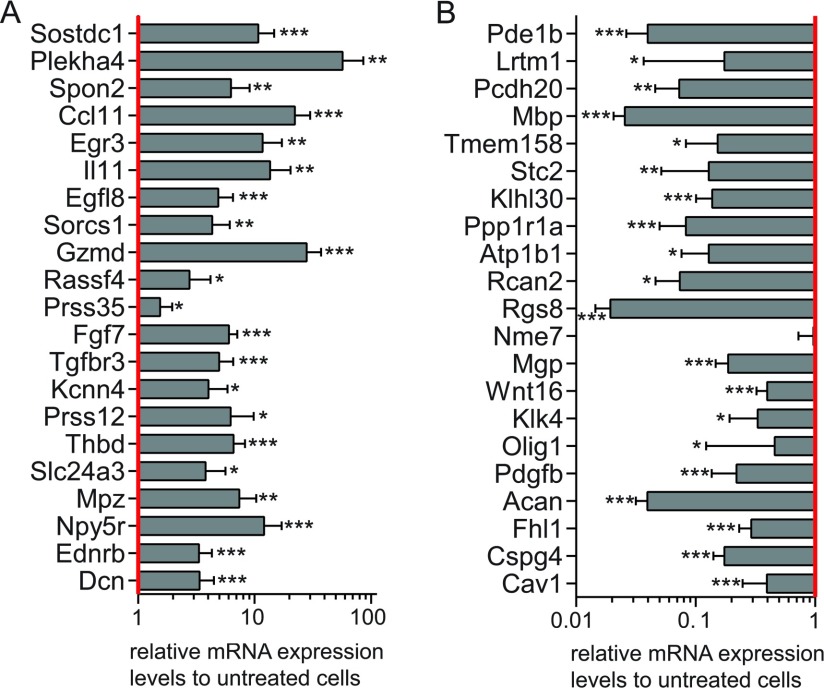
Differential gene expression upon forskolin treatment The differential expression of the strongest increased (A) and decreased (B) gene transcripts was validated by qRT–PCR in treated compared with untreated primary mouse Schwann cells. Data were normalized to the expression of 60s. The columns represent the mean value of 12 experimental samples, and the error bars indicate the S.D. *: *P*≤0.005, **: *P*≤0.0001, ***: *P*≤0.00001. Raw data are provided as Supplementary Table S3 (available at http://www.asnneuro.org/an/006/an006e142add.htm).

To further investigate the effect of forskolin on transcriptional regulation in cultured mouse Schwann cells, microarray data were analyzed more stringently using an FDR-adjusted *P*-value of <0.05. This study revealed that forskolin treatment resulted in increased expression of 330 transcripts by at least 1.5-fold, and in decreased expression of 305 transcripts (Supplementary Table S2; available at http://www.asnneuro.org/an/006/an006e142add.htm). Among the 25 strongest induced genes, Mpz was the solely typical so far known myelin-related gene (Table 2A). Strong transcriptional induction was detected for the sclerostin domain containing 1 (Sostdc1), the pleckstrin homology domain containing family A (Plekha4) and the ECM (extracellular matrix) protein spondin 2 (Spon2). Furthermore, increased transcription was detected for the transcription factor Egr3, as already stated before (Table 1A), the fibro-blast growth factor 7 (Fgf7), the endothelin receptor type B (Ednrb) and the proteoglycan decorin (Dcn), to name a few. Strongest down-regulation upon forskolin treatment was detected for the mRNA expression levels of protocadherin 20 (Pcdh20, also known as Pcdh13), phosphodiesterase 1B (Pde1b) and leucine-rich repeats and transmembrane domains 1 (Lrtm1), each represented by two transcripts (Table 2B). Olig1 was the only transcription factor identified among the 25 strongest reduced transcripts. Strong transcriptional reduction was also identified for the chondroitin sulfate proteoglycan 4 [Cspg4, better known as neuron-glial antigen 2 (NG2)], the proteoglycan aggrecan (Acan), the secreted matrix Gla protein (Mgp) and the platelet-derived growth factor subunit B (Pdgfb). qRT–PCR of these highly differentially expressed transcripts validated our microarray for the one which were significantly increased upon forskolin treatment (Figure 1A) as well as for the ones which were down-regulated upon forskolin treatment with the sole exception of Nme7 (Figure 1B).

**Table 2:** The strongest forskolin-dependent differentially regulated transcripts Microarray data of Schwann cells cultured in the presence or absence of forskolin were analyzed using a two-way ANOVA with an FDR-adjusted *P-*value < 0.05. The 25 transcripts with the strongest increased (A) or reduced (B) mRNA expression levels in treated Schwann cells are itemized, and their putative role in Schwann cells is indicated. n.d.: not determined. ^1)^Vigo et al., 2005 ^2)^Yang et al., 1998 ^3)^Gao et al., 2007 ^4)^Napoli et al., 2012 ^5)^Srinivasan et al., 2012 ^6)^Thomas and de Vries, 2007 ^7)^Wolfer et al., 2001 ^8)^Wilkins et al., 1997 ^9)^Hanemann et al., 1993 ^10)^ D. Schmid, T. Zeis, M. Sobrio and N. Schaeren-Wiemers, unpublished work ^11)^Arthur-Farraj et al., 2012 ^12)^Ogata et al,. 2004 ^13)^Jiang et al., 2013 ^14)^Monje et al., 2009 ^15)^Afshari et al., 2010 ^16)^Jesuraj et al., 2012 ^17)^Schneider et al., 2001 ^18)^Rezajooi et al., 2004 ^19)^Mikol et al., 1999 ^20)^Mikol et al., 2002 ^21)^Tan et al., 2003

	Entrez ID	Official name	Putative role in Schwann cells	Fold change	Ratio 20 to 0 μM	*P-*value
**A**	**Sostdc1**	Sclerostin domain containing 1, ectodin, wise	n.d.	35.54	35.54	< 0.0001
	**Plekha4**	Pleckstrin homology domain containing, family A	Other pleckstrin homology domain containing proteins are known in Schwann cells	17.53	17.53	< 0.0001
	**Spon2**	Spondin 2, extracellular matrix protein, M-spondin	Down-regulated in a PMP22-overexpressing rat model^1^	14.12	14.12	0.0011
	**Ccl11**	Chemokine (C–C motif) ligand 11, eotaxin	Suggested to be expressed in Schwann cells^2^	10.68	10.68	< 0.0001
	**Egr3**	Early growth response 3	Modulates p75^NTR^ expression together with Egr1^3^	7.26	7.26	< 0.0001
	**Il11**	Interleukin 11; adipogenesis inhibitory factor (AGIF)	Increased following Raf activation^4^	7.24	7.24	< 0.0001
	**Ccl11**	Chemokine (C-C motif) ligand 11, eotaxin	Suggested to be expressed in Schwann cells^2^	6.20	6.20	0.0002
	**Egfl8**	EGF-like-domain, multiple 8	Activated by Sox10, down-regulated by Egr2^5^	6.19	6.19	0.0001
	**Sorcs1**	VPS10 domain receptor protein SORCS 1, sortilin-related receptor CNS expressed 1	n.d.	5.68	5.68	< 0.0001
	**Egfl8**	EGF-like domain, multiple 8	Activated by Sox10, down-regulated by Egr2^5^	5.04	5.04	0.0001
	**Gzmd**	Granzyme D	n.d.	4.95	4.95	0.0024
	**Rassf4**	Ras association (RalGDS/AF-6) domain family member 4	n.d.	4.34	4.34	0.0045
	**Prss35**	Protease, serine, 35	n.d.	4.18	4.18	0.0003
	**Plekha4**	Pleckstrin homology domain containing, family A	Other pleckstrin homology domain containing proteins are known in Schwann cells	4.15	4.15	< 0.0001
	**Fgf7**	Fibroblast growth factor 7, heparin-binding growth factor, keratinocyte growth factor	Expressed in Schwann cells^6^	3.78	3.78	0.0004
	**Tgfbr3**	Transforming growth factor, beta receptor III, betaglycan	Expressed in Schwann cells^6^	3.72	3.72	< 0.0001
	**Kcnn4**	Potassium intermediate/small conductance calcium-activated channel	n.d.	3.62	3.62	< 0.0001
	**Prss12**	Protease, serine 12, neurotrypsin, motopsin	Expressed in Schwann cell precursor, suggested role in Schwann cell differentiation^7^	3.61	3.61	< 0.0001
	**Thbd**	Thrombomodulin, fetomodulin	n.d.	3.59	3.59	0.0001
	**Slc24a3**	Solute carrier family 24 (sodium/potassium/calcium exchanger), member 3	n.d.	3.53	3.53	< 0.0001
	**Mpz**	Myelin protein zero	Expressed in myelinating Schwann cells, marker for differentiation	3.41	3.41	0.0001
	**Npy5r**	Neuropeptide Y receptor Y5	n.d.	3.31	3.31	< 0.0001
	**Ednrb**	Endothelin receptor type B	Endothelin receptors were shown to be coupled to adenylyl cyclase in immortalized Schwann cells^8^	3.25	3.25	< 0.0001
	**Dcn**	Decorin (proteoglycan)	Expressed in Schwann cells; increased from E14 to E18^9^	3.16	3.16	0.0007
	**Prss12**	Protease, serine, 12 neurotrypsin (motopsin)	Expressed in Schwann cell precursor, suggested role in Schwann cell differentiation^7^	3.16	3.16	< 0.0001
**B**	**Pcdh20**	Protocadherin 20, protocadherin 13	n.d.	−14.02	0.07	< 0.0001
	**Pde1b**	Phosphodiesterase 1B, Ca^2+^-calmodulin dependent	n.d.	−12.05	0.08	< 0.0001
	**Lrtm1**	Leucine-rich repeats and transmembrane domains 1	n.d.	−11.60	0.09	< 0.0001
	**Pde1b**	Phosphodiesterase 1B, Ca^2+^-calmodulin dependent	n.d.	−11.30	0.09	< 0.0001
	**Lrtm1**	Leucine-rich repeats and transmembrane domains 1	n.d.	−10.92	0.09	< 0.0001
	**Pcdh20**	Protocadherin 20	n.d.	−10.07	0.10	< 0.0001
	**Mbp**	Myelin basic protein	Sequence codes for Mbp variants 1-7 including Golli-Mbp, having a distinct function than classical Mbp	−9.98	0.10	< 0.0001
	**Tmem158**	Transmembrane protein 158, ras-induced senescence 1 (ris1)	n.d.	−8.85	0.11	0.0002
	**Stc2**	Stanniocalcin 2, mustc2	n.d.	−8.82	0.11	< 0.0001
	**Klhl30**	Kelch-like 30	n.d.	−7.04	0.14	< 0.0001
	**Ppp1r1a**	Protein phosphatase 1, regulatory (inhibitor) subunit 1A	n.d.	−5.83	0.17	< 0.0001
	**Atp1b1**	ATPase, Na^+^/K^+^ transporting, beta 1 polypeptide	n.d.	−4.97	0.20	< 0.0001
	**Rcan2**	Regulator of calcineurin 2, calcipressin-2, MCIP2	n.d.	−4.87	0.21	< 0.0001
	**Rgs8**	Regulator of G-protein signaling 8	n.d.	−4.61	0.22	< 0.0001
	**Nme7**	ME/NM23 family member 7, non-metastatic cells 7, Nucleoside diphosphate kinase 7	n.d.	−4.12	0.24	< 0.0001
	**Mgp**	Matrix Gla protein	n.d.	−4.01	0.25	0.0004
	**Wnt16**	Wingless-related MMTV integration site 16	Increased in MAL-overexpressing Schwann cells^10^	−4.01	0.25	< 0.0001
	**Klk4**	Kallikrein related-peptidase 4 (prostase, enamel matrix, prostate)	n.d.	−3.95	0.25	< 0.0001
	**Olig1**	Oligodendrocyte transcription factor 1	Strong cJun-dependent activation in denervated Schwann cells^11^	−3.94	0.25	0.0009
	**Pdgfb**	Platelet-derived growth factor, B polypeptide	Pdgf suppresses exression of myelin-related proteins^12^ and promotes cell proliferation^13, 14^	−3.84	0.26	0.0003
	**Acan**	Aggrecan, Cspg1	Schwann cell migration is inhibited by astrocyte-produced aggrecan^15^	−3.72	0.27	< 0.0001
	**Fhl1**	Four and a half LIM domains 1	Up-regulated in the motor branch of the femoral nerve compared to the sensory branch^16^	−3.64	0.27	< 0.0001
	**Wnt16**	Wingless-related MMTV integration site 16	Increased in MAL-overexpressing Schwann cells^10^	−3.56	0.28	< 0.0001
	**Cspg4**	Chondroitin sulfate proteoglycan 4, neuron-glial antigen 2 (NG2), AN2	Expressed in precursor, immature and nonmyelinating Schwann cells^18^, up-regulated in regenerating PNS^18^	−3.52	0.28	< 0.0001
	**Cav1**	Caveolin 1	Increased during myelinating and decreased after axotomy^19, 20^, can regulate the signaling through ErbB2^21^	−3.49	0.29	< 0.0001

In summary, cAMP elevation led to differential expression of more than 600 transcripts in primary mouse Schwann cell cultures. Among the 25 strongest induced genes, Mpz was the only well-known myelin-related gene, disclosing the possibility that new genes important for Schwann cell differentiation were identified by this microarray analysis.

### Olig1 expression in the PNS

**Figure 2:**
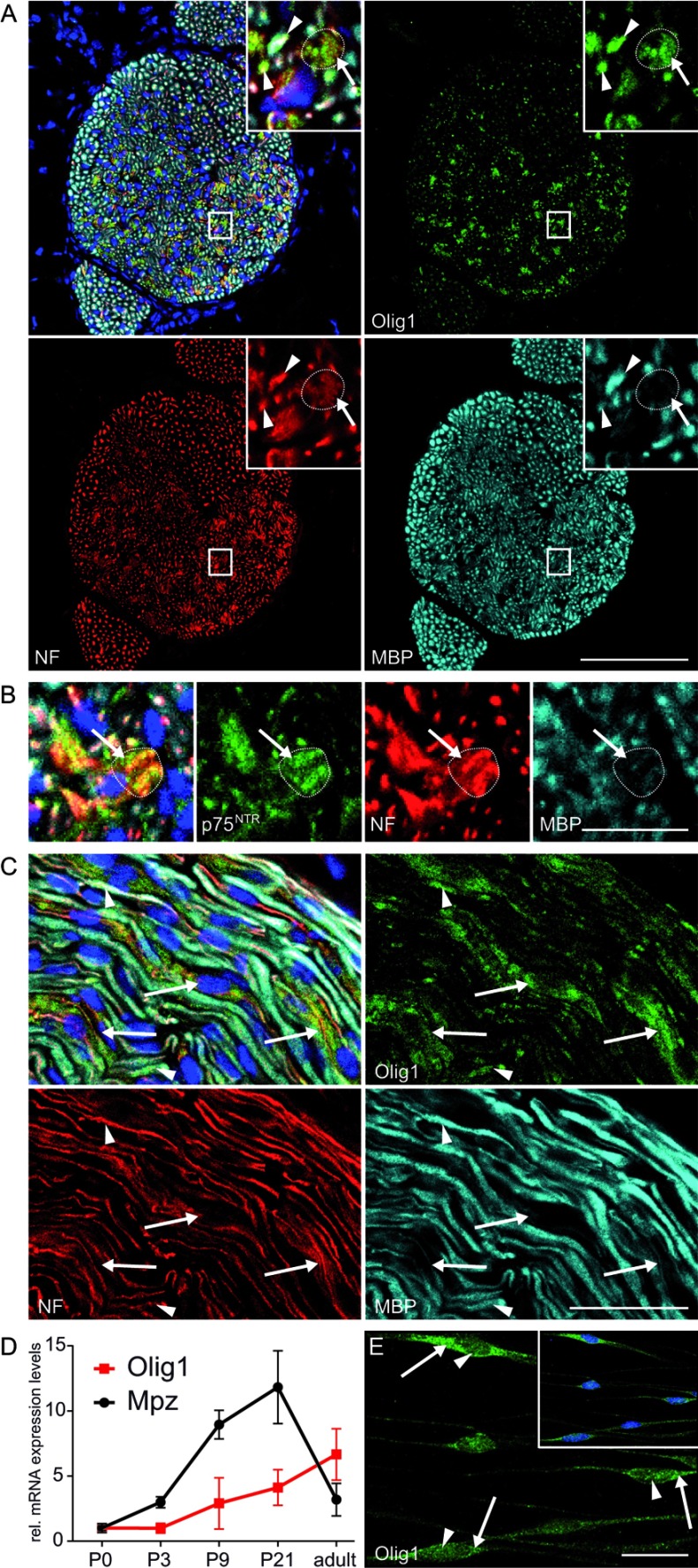
Expression of Olig1 in sciatic nerves and cultured Schwann cells Immunofluorescent stainings of transversal (A) and longitudinal (C) tissue sections of sciatic nerves from P7 mice revealed Olig1 immunofluorescence in Remak bundles (arrows), identified by a bundle of unmyelinated (MBP-negative) small diameter axons, and in a subset of myelinating Schwann cells (arrowheads). (B) Remak bundle localization was confirmed by the expression of p75^NTR^. (D) A progressive increase of Olig1 mRNA expression levels was detected during peripheral nerve development by qRT–PCR. Data were normalized to the expression of 60s, and values at P0 were set to 1. Each data point represent the mean value of at least eight experimental samples, and the error bars indicate the S.D. (E) *In vitro*, Olig1 expression was predominantly detected in the cytoplasm of cultured Schwann cells (E, arrows), whereas nuclear staining was significantly weaker (E, arrowheads). NF: neurofilament. Bar: A: 100 μm; B, C, E: 20 μm.

The transcription factor Olig1 was identified to be strongly down-regulated in forskolin-treated mouse Schwann cells. Although Olig1 is known to play an important functional role in differentiation of oligodendrocyte precursor cells (Li et al., 2007), its expression in the Schwann cell lineage is not known. For this reason, the expression of Olig1 protein was investigated on sciatic nerves of P7 mice. Colocalization analysis revealed Olig1 immunofluorescent signal primarily in MBP-negative areas consisting of small diameter axons identified by neurofilament (Figure 2B, inset and Figure 2D, arrows). These areas are reminiscent of Remak bundles evident by the expression of p75^NTR^, a marker for nonmyelinating Schwann cells (Figure 2B and Supplementary Figure S2; available at http://www.asnneuro.org/an/006/an006e142add.htm). In addition, also a subset of myelinating Schwann cells were identified to be Olig1 positive (Figures 2A and 2C, arrowheads). To determine Olig1 transcription during peripheral nerve development, its mRNA expression levels were investigated by qRT-PCR in sciatic nerves at P0, P3, P9, P21 and in adult mice (Figure 2D). Expression analysis of Olig1 mRNA levels during peripheral nerve development revealed progressively increased transcription correlating with Schwann cell maturation. In contrast to Mpz, Olig1 mRNA expression levels remained high in adult nerves. Confocal immunofluorescence microscopy identified Olig1 localization in the cytoplasm as well as in the nucleus of Schwann cells (Figure 2E). Quantification of the average immunofluorescent signal revealed a significant higher intensity in the cytoplasm compared with the nucleus (results not shown). From our analysis, we conclude that Olig1 is expressed in Schwann cells although at lower levels than in the CNS (central nervous system) (results not shown).

### Analysis of possible forskolin-dependent upstream regulators

Putative forskolin-dependent upstream regulators were identified by IPA (Ingenuity Pathway Analysis), based on the highly significantly regulated transcripts (Supplementary Table S2). The highest significantly proposed upstream regulator was identified as ‘forskolin’ (Table 3). Furthermore, three kinases playing a role in regulating the NF-κB (nuclear factor κB) pathway were suggested as upstream regulators, namely the inhibitor of NF-κB kinase subunit

**Table 3:** Investigation of putative upstream regulators Differentially expressed transcripts in differentiated Schwann cells were analyzed in respect to their potential upstream regulators.

Upstream regulator	Common name	Predicted activation state	Activation *z-*score	*P-*value of overlap
**Forskolin**		Activated	2.508	3.90×10^−15^
**CHUK**	Inhibitor of nuclear factor kappa-B kinase subunit α	Activated	2.387	4.83×10^−15^
**IKBKG**	Inhibitor of kappaB kinase subunit γ	Activated	2.982	1.61×10^−13^
**IKBKB**	Inhibitor of kappaB kinase subunit β	Activated	2.985	1.21×10^−10^
STAT3	Signal transducer and activator of transcription 3	Activated	2.462	8.81×10^−7^
CEBPB	C/EBP-beta, NF-IL6	Activated	2.201	5.81×10^−6^
**SOX10**	SRY-box containing gene 10	Activated	2.200	1.57×10^−5^
HOXC8	Homeobox C8	Activated	2.000	6.41×10^−3^
Tnf (family)	Tumor necrosis factor	Activated	2.132	9.84×10^−3^
HOXC6	Homeobox C6	Activated	2.000	1.51×10^−2^
ZNF217	Zinc finger protein 217	Activated	2.236	3.36×10^−2^
PTPRJ	Protein tyrosine phosphatase, receptor type, J	Activated	2.000	4.33×10^−2^
**NOTCH1**	Notch1	Inhibited	−2.318	8.05×10^−6^
SRF	Serum response factor	Inhibited	−2.668	1.12×10^−5^
TGFB3	Transforming growth factor, β 3	Inhibited	−2.559	3.31×10^−5^
KLF4	Kruppel-like factor 4	Inhibited	−2.599	5.93×10^−4^
Nfat (family)	Nuclear factor of activated T cells	Inhibited	−2.121	6.60×10^−4^
LYN	Yamaguchi sarcoma viral (v-yes-1) oncogene homolog	Inhibited	−2.186	4.21×10^−3^
**RAC1**	RAS-related C3 botulinum substrate 1	Inhibited	−2.173	5.11×10^−3^
**MAP2K1/2**	Mek1/2	Inhibited	−2.177	9.43×10^−3^
MKL1	MKL (megakaryoblastic leukemia)/myocardin-like 1	Inhibited	−2.160	1.26×10^−2^
SFTPA1	Surfactant associated protein A1	Inhibited	−2.200	3.19×10^−2^
IKZF1	IKAROS family zinc finger 1	Inhibited	−2.433	3.92×10^−2^

α (CHUK), the I-κB kinase subunit γ (IKBKG) and the I-κB kinase subunit β (IKBKB). In addition, the signal transducer and activator of transcription 3 (Stat3) and Sox10 were proposed to be involved in the regulation of some of the differentially expressed genes. Notch1 was proposed as the most significant inhibited upstream regulator, well in line with the negative effect of Notch signaling on peripheral myelination (Woodhoo et al., 2009). In addition, inhibitory modulation was proposed for the small GTP-binding protein Rac1 and the MAPK-kinase MEK 1 and 2 (MAP2K1/2).

An important question to solve is whether there are putative transcription factor binding sites in promoter regions of co-regulated genes, allowing to identify transcription factors that might cause some of the observed alterations. Using TransFind, a web-based software tool, the affinity of a transcription factor to the putative promoters of the genes (−300 bp upstream to +100 bp downstream of transcription start site) was predicted *in silico* (Kielbasa et al., 2010). First, genes with induced mRNA expression levels due to forskolin treatment were analyzed (Table 4A). The most significant prediction was for the transcription factor matrix of the KROX family, containing Krox24 (Egr1), Krox20 (Egr2), Egr3 and NGFI-C (Egr4) and the distally related Wilms’ tumor 1 (Wt1) (Chavrier et al., 1988; Lemaire et al., 1988; Call et al., 1990; Gessler et al., 1990; Patwardhan et al., 1991; Crosby et al., 1992). In addition, genes with a binding site for the transcription factors spermatogenic leucine zipper 1 (Spz1) and Wt1 were significantly overrepresented in forskolin-induced genes. We could further identify that several genes with reduced mRNA expression levels in treated Schwann cells contain a binding site for the transcription factor HEB (also known as transcription factor 12, Tcf12) or the myocyte enhancer factor-2 (MEF-2) (Table 4B).

**Table 4:** Promoter analysis to investigate significantly enriched transcription factor binding sites Three putative transcription factor binding sites could be identified for gene transcripts increased due to forskolin treatment (A), whereas two putative binding sites could be detected for decreased gene transcripts (B). TF: Transcription factor. ^a)^ number and percentage of genes that contain specific transcription factor-binding site among submitted transcripts; ^b)^ number and percentage of genes that contain specific transcription factor binding site among all genes.

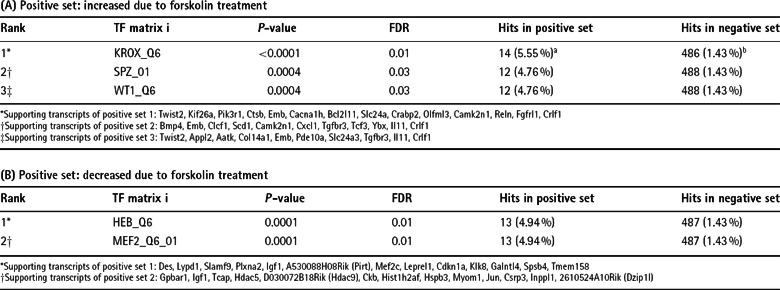

### GO-annotations in differentiated Schwann cells

**Figure 3:**
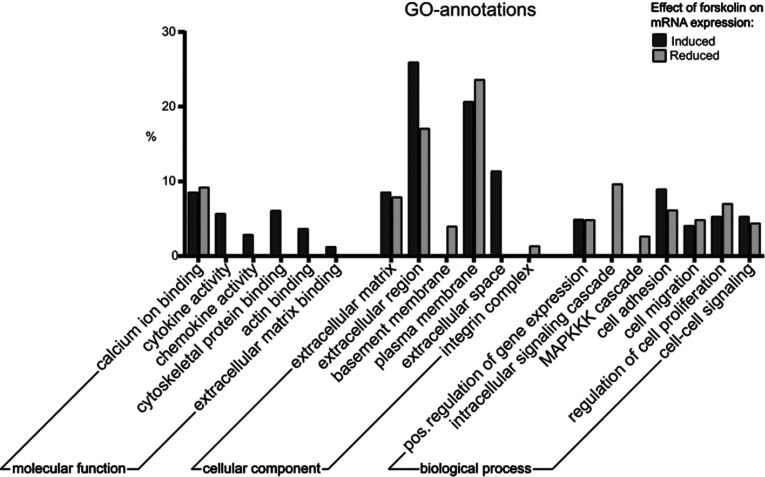
GO-annotations of differentially expressed genes due to forskolin treatment Analysis of molecular functions revealed that several transcripts increased with forskolin are associated with cytoskeletal protein and actin binding. Both sets of transcripts either increased or decreased due to forskolin manifested association with the cellular component of ECM and with the plasma membrane. The term of basement membrane and integrin complex was exclusively enriched in forskolin-reduced transcripts. GO-annotation analysis of transcripts decreased with forskolin showed an enrichment of genes implicated in intracellular signaling cascade and in the MAPKK cascade. Raw data are provided as Supplementary Table S4 (available at http://www.asnneuro.org/an/006/an006e142add.htm).

The usage of GO (gene ontology) annotations allows analyzing differentially expressed transcripts by means of a controlled vocabulary, in respect to the categories of cellular components, molecular functions and biological processes (Figure 3 and Supplementary Table S4; available at http://www.asnneuro.org/an/006/an006e142add.htm). Ana-lysis of molecular functions revealed that forskolin-induced transcripts were associated with cytoskeletal protein binding, actin binding and ECM binding, as well as with cytokine and chemokine activity. Investigation of GO-annotations on cellular components revealed a high association of both sets of transcripts with the ECM, the extracellular region and the plasma membrane. Furthermore, genes with reduced mRNA expression levels in treated cells showed enrichment for the annotations basement membrane and integrin complex. Analysis of the category ‘biological processes’ revealed that increased and decreased transcripts were often associated with cell adhesion, migration and proliferation, as well as with cell–cell signaling. The term of MAPKKK (MAPK kinase kinase) cascade, which is implicated in Schwann cell dedifferentiation (Harrisingh et al., 2004), was detected exclusively in the set of transcripts with reduced expression due to forskolin treatment.

### Pathway analysis implicated in Schwann cell differentiation

To identify putative signaling cascades in forskolin-dependent differentially expressed transcripts, the KEGG (Kyoto Encyclopedia of Genes and Genomes) pathway was investigated using DAVID (Table 5). Also this analysis revealed that several forskolin-induced gene transcripts were associated with the ECM–receptor interaction (Table 5A). Pathway analysis of transcripts, which were reduced upon elevation of cAMP levels revealed the strongest enrichment for the focal adhesion pathway (Table 5B). In line with induced transcripts, ECM–receptor interaction was also proposed as a putative pathway for the set of genes with decreased mRNA expression levels in treated Schwann cells. Furthermore, the MAPK (MAP-kinase) signaling pathway was identified to be overrepresented in this set of transcripts. Based on the pathway analysis, we conclude that a number of differentially regulated transcripts were associated with the ECM–receptor interaction as well as with the focal adhesion pathway, implicating that a major effect of forskolin might be the modulation of the ECM and the cytoskeleton.

**Table 5:** KEGG pathway analysis of differentially expressed transcripts in primary mouse Schwann cell cultures Gene with induced (A) and those with reduced mRNA expression levels (B) due to forskolin treatment were analyzed using the software DAVID. Both sets manifested enrichment for the ECM–receptor interaction and for focal adhesion. % of total submitted genes (294 for A, 242 for B).

(A) Set of genes with increased mRNA expression levels due to forskolin			
Identification	Pathway	%	*P*-value
mmu04060	Cytokine–cytokine receptor interaction	5.10	0.00020
mmu04512	ECM–receptor interaction	2.38	0.00446
mmu04621	NOD-like receptor signaling pathway	2.04	0.00528
mmu04360	Axon guidance	2.72	0.01129
mmu04350	TGF-beta signaling pathway	2.04	0.02338
mmu04510	Focal adhesion	3.06	0.03265
(B) Set of genes with decreased mRNA expression levels due to forskolin			
Identification	Pathway	%	*P*-value
mmu04510	Focal adhesion	7.02	<0.00001
mmu04512	ECM–receptor interaction	3.31	0.00028
mmu04115	p53 signaling pathway	2.89	0.00060
mmu04810	Regulation of actin cytoskeleton	3.72	0.01811
mmu04012	ErbB signaling pathway	2.07	0.04833
mmu04010	MAPK signaling pathway	3.72	0.05746

### Forskolin-dependent regulation of components of the ECM

**Figure 4:**
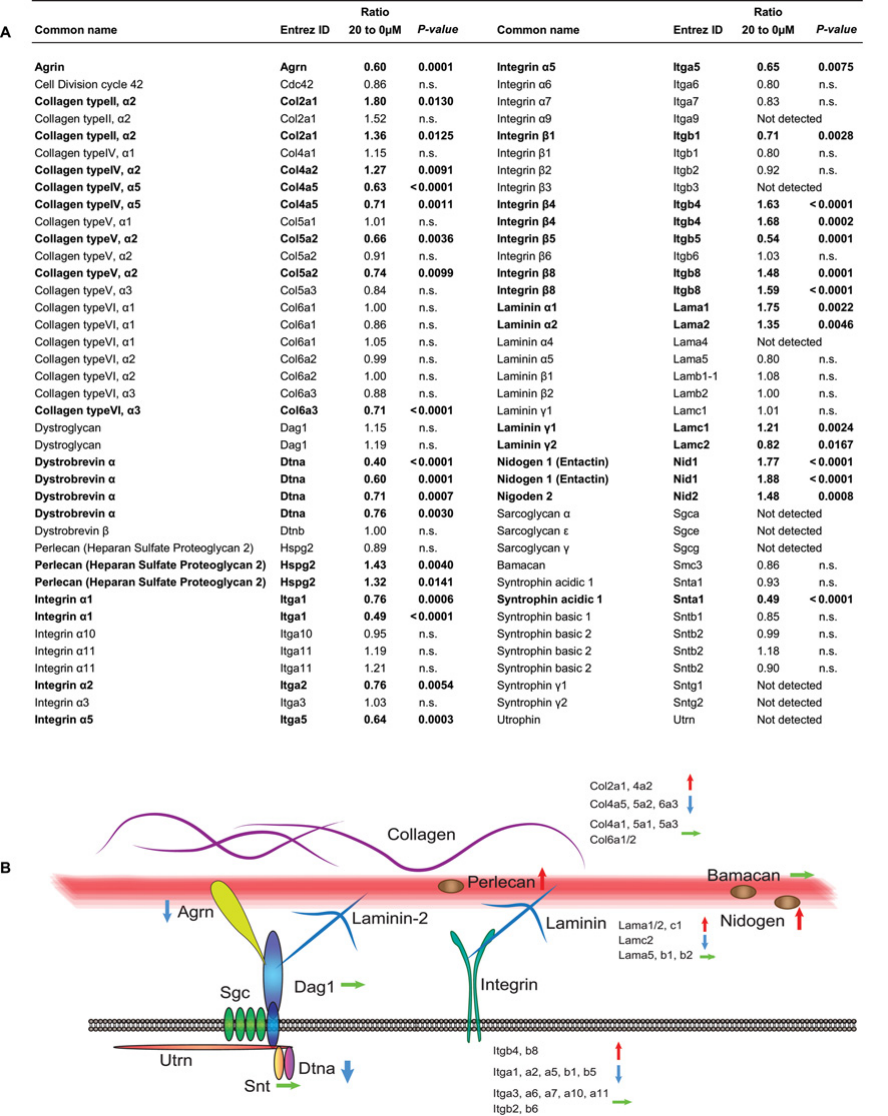
Effect of forskolin treatment on mRNA expression levels of known components of the ECM in Schwann cells (A) Data analysis of the microarray was performed by a two-way ANOVA, and unadjusted *P*-values <0.01 were accounted as significant. Forskolin had a regulatory effect on the majority of investigated ECM-associated genes. n.s.: not significant. (B) Schematic illustration of components of the ECM and the basal lamina in Schwann cells. The effect of forskolin on gene expression of selected genes associated with the ECM and the basal lamina (red line) was shown by blue (reduced), red (increased) or green (unaltered) arrows.

To determine the effect of elevated cAMP levels by forskolin on transcriptional regulation of components of the ECM and the basal lamina, a list of selected genes associated with the ECM was compiled and schematic illustrated (Figure 4). Most of the investigated components of the basal lamina showed differentially expressed mRNA expression levels upon forskolin treatment of cultured Schwann cells. One of the strongest reductions could be detected for α-dystrobrevin (Dtna), a member of the DGC (dystrophin–glycoprotein complex). Furthermore, the transcription of syntrophin acidic 1 (Snta1), also a component of this complex, was significantly reduced upon forskolin treatment, although expression levels of the other syntrophin isoforms were not changed. The DGC is linked to the basal lamina by interaction with agrin (Agrn), whose mRNA expression levels were also reduced in treated Schwann cells, or with laminin. A reduced expression was identified for laminin γ2 (Lamc2), whereas increased expression levels were detected for laminin α1, α2 (Lama1, Lama2) and laminin γ1 (Lamc1) upon treatment. Investigation of the laminin receptor integrin manifested that forskolin treatment reduced the transcription of integrins such as the integrin α1, α2, α5, β1 and β5 (Itga1, Itga2, Itga5, Itgb1 and Itgb5) in primary mouse Schwann cells. In contrast, forskolin treatment induced the transcription of integrin β4 and β8 (Itgb4 and Itgb8), in agreement with a study reporting elevated integrin β4 expression during development and upon forskolin treatment in rat Schwann cells (Feltri et al., 1994). Collagen fibers are another major component of the ECM. Elevation of cAMP levels by forskolin resulted in induced transcription of collagen type II α1 (Col2a1), in line with a previous report on rat Schwann cells (D’Antonio et al., 2006), and collagen type IV α2 (Col4a2). Forskolin treatment led to reduced transcription of collagen type IV α5 (Col4a5), collagen type V α2 (Col5a2) and of collagen type VI α3 (Col6a3). Additional analysis of basal lamina components revealed significantly increased mRNA expression levels of nidogen1 (entactin, Nid1), nidogen2 (entactin 2, Nid2) and of the proteoglycan perlecan (Hspg2).

From these data, we conclude that forskolin treatment has a strong impact on transcriptional regulation of a variety of ECM-associated genes in cultured mouse Schwann cells, probably reflecting the morphological changes occurring upon Schwann cell differentiation.

### Correlation analysis between *in vivo* and *in vitro* samples

**Figure 5:**
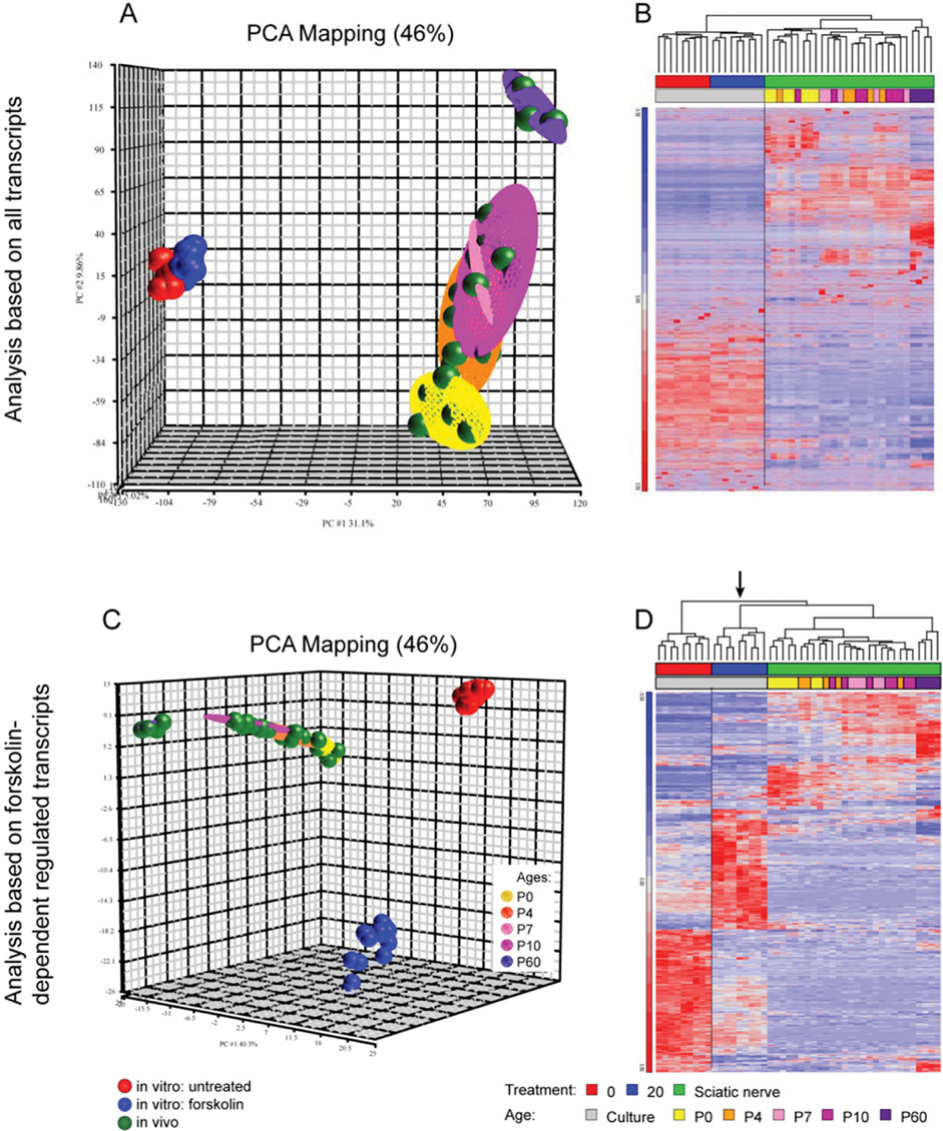
Comparison analysis of gene expression between primary mouse Schwann cell cultures and developing sciatic nerve samples (A, B) Analysis of the whole-genome revealed distinct gene transcription between *in vivo* and *in vitro*, illustrated by PCA (A). A well-defined cluster can be depicted of developing nerve samples, whereas P60 form an individual cluster. Distinct expression between primary Schwann cell cultures and *in vivo* samples could be confirmed by a heat map analysis, indicated by the dendrogram (B). (C, D) Analysis based on only forskolin-dependent differentially expressed transcripts resulted in distinct clusters between treated and untreated Schwann cells, as well as between *in vivo* and *in vitro* (C). Heat map analysis revealed that forskolin-treated Schwann cells associate within the same branch as the samples derived from the nerve tissues (D, arrow).

Elevation of intracellular cAMP by forskolin is often used as an *in vitro* model for Schwann cell differentiation. Therefore the expression data from naive and forskolin-treated Schwann cells and from sciatic nerve tissues taken from P0, P4, P7, P10 and P60 mice were examined by a PCA to visualize similarities or differences between the experimental samples (Figure 5A). Well-defined clusters could be identified for both untreated and treated Schwann cell cultures (Figure 5A, red and blue dots, respectively). Additional distinct clusters could be detected for samples of the different developmental time points of peripheral nerves (Figure 5A, green dots). The PCA for the developmental *in vivo* samples at P0, P4, P7 and P10 showed that the time points closely neighbor each other. P60 nerve samples formed an individual cluster, reflecting that their expression is distinct. The PCA also illustrated that the expression pattern identified in cultured Schwann cells did not overlap with those of the peripheral nerve tissues, which is reflected by the dendrogram analysis of hierarchical clustering (Figure 5B).

The effect on elevated cAMP levels on the Schwann cell lineage was further investigated by analyzing only transcripts which were differentially expressed with an FDR-adjusted *P*-value <0.05 due to forskolin treatment (Figures 5C and 5D). By PCA, distinct clusters could be visualized for samples of treated or untreated Schwann cell cultures (Figure 5C). Hierarchical cluster analysis revealed further that forskolin-treated Schwann cell samples associate within the same branch as samples derived from the nerve tissues (Figure 5D, arrow), indicating some corresponding expression pattern. Still, the majority of differentially expressed transcripts elicited by forskolin treatment do not resemble the *in vivo* situation.

## DISCUSSION

Accurate peripheral myelination depends on a variety of signals and growth factors. Elevation of intracellular cAMP levels by either db-(dibutyryl-)cAMP or forskolin is the classically used stimulation assay to reproduce some of the aspects of Schwann cell differentiation *in vitro*, reflected by induced expression of myelin-related genes (Monuki et al., 1989; Morgan et al., 1991; Parkinson et al., 2003; Schworer et al., 2003; Monje et al., 2009). The effect of forskolin treatment on primary mouse Schwann cells was analyzed by a comprehensive microarray study. Comparison between our microarray data and a study on rat Schwann cells treated with forskolin revealed a number of overlaps such as increased expression of inositol triphosphate receptor subtype 3 (Itpr3), laminin α2 and γ1 (Lama2, Lamc1) and reduced expression of agrin (Agrn), fibroblast growth factor 5 (Fgf5) and focal adhesion kinase pp125 (Ptk2) (Schworer et al., 2003). In addition to the myelin-related genes known to be regulated by forskolin *in vitro*, we identified many so far disregarded genes to be expressed by cultured Schwann cells. The generated database and interactive search tool for genes of interest provides a new insight into the molecular mechanisms of Schwann cell differentiation (Interactive Excel file; available at http://www.asnneuro.org/an/006/an006e142add.htm).

First, we focused on genes that were reported to be expressed at a distinct stage of the Schwann cell lineage (Supplementary Figure S1). In line with previous studies, we observed induced transcription of genes expressed in differentiated Schwann cells, such as the myelin-related genes Mpz and Pmp22 (Lemke and Chao, 1988; Morgan et al., 1991; Schworer et al., 2003; Monje et al., 2009). However, no increase on mRNA expression levels could be detected for Mag. This observation is in contrast to reports on rat Schwann cells showing increased protein levels upon db-cAMP addition (Monje et al., 2009; Monje et al., 2010), respectively, increased Mag transcription after forskolin treatment (Ogata et al., 2004). This discrepancy of Mag expression compared with published literature might be explained by the fact that Schwann cells derived from mice were investigated in our study, compared with Schwann cells derived from rats in the other studies. Indeed, we found no report analyzing Mag transcription upon forskolin treatment in primary mouse Schwann cell cultures. We suggest that the effect of forskolin in respect to Mag transcription might be distinct between rat and mouse Schwann cells.

Induction of myelin-related gene transcripts was also reported in cultured Schwann cells upon adenoviral infection with an Egr2-expressing construct (Nagarajan et al., 2001). In comparison with their study, we also identified a down-regulation of genes expressed in neural crest cells, Schwann cell precursors and immature Schwann cells, such as the transcription factors AP2α (Tcfap2a) and cJun or the adhesion proteins Cdh2 (NCad) and Cdh19. These observations suggest that Egr2-dependent differentiation drives the Schwann cells predominantly into the myelinating phenotype, whereas treatment with forskolin additionally leads to down-regulation of gene transcripts expressed at earlier stages of the lineage.

Upon forskolin treatment, we identified significantly increased expression for the transcription factor Egr1 (Krox24), a major transcription factor in nonmyelinating Schwann cells. The induced transcription might be due to the Egr1 promoter containing a CRE (cAMP-response element) (reviewed in Thiel et al., 2010). Furthermore, increased transcriptional activity of Egr1 by forskolin is in line with detected transactivation of Egr1 by CREB signaling in gonadotophs (Mayer et al., 2008; Mayer and Thiel, 2009). Besides Egr1, also the related transcription factor Egr3 was significantly increased by around 7-fold in treated Schwann cells. Enforced expression of both Egr1 and Egr3 by adenoviral infections led to increased p75^NTR^ transcription in immortalized rat Schwann cells (Gao et al., 2007), in contrast to our data of reduced p75^NTR^ expression despite increased Egr1 and Egr3 mRNA expression levels. However, the forskolin-induced increase of Egr1 transcription was modest compared with enforced expression by adenoviral constructs, speculating that a certain expression level and/or a distinct stoichiometry of Egr1 and Egr3 is required to modulate p75^NTR^ expression. Analysis of tyrosine kinase receptors revealed that transcripts coding for ErbB2, TrkB and TrkC were increased in treated Schwann cells, suggesting that forskolin treatment not only activates CREB signaling but also might influence other intracellular signaling pathways mediated by tyrosine kinase receptors in Schwann cells as previously suggested (Stewart et al., 1996; Kim et al., 1997; Cohen and Frame, 2001; Grimes and Jope, 2001; Ogata et al., 2004; Monje et al., 2006; Monje et al., 2010).

### Olig1 as a new transcription factor in Schwann cells

Among the 25 gene transcripts, which were strongly decreased in forskolin-treated Schwann cells, we identified the transcription factor Olig1. In the CNS, Olig1 is essential for the differentiation of oligodendrocyte precursor cells into mature oligodendrocytes (Xin et al., 2005; Li et al., 2007). In the oligodendrocytes lineage, Olig1 activates Mbp transcription by interaction with the transcription factor Sox10 (Li et al., 2007), which is also expressed throughout the entire Schwann cell lineage. However, the role of Olig1 in the PNS is not known yet. Although investigations of Olig1-deficient mice did not reveal major abnormalities of the PNS (Charles D. Stiles, personal communication), a functional role of Olig1 during peripheral nerve development or in regeneration cannot be excluded. In cultured mouse Schwann cells, a significantly higher Olig1 expression was detected in the cytoplasm compared with the nucleus. In the CNS, Olig1 was also localized in the cytoplasm of oligodendrocytes, whereas nuclear localization was only detected for a short time period during early development (Arnett et al., 2004; Othman et al., 2011). The cytoplasmic function of Olig1 is not yet entirely understood, but it was demonstrated to be crucial for elaboration of cell processes and membrane expansions in oligodendrocytes precursor cells (Niu et al., 2012). Since glial development in the PNS precedes the one in the CNS, Olig1 expression in the nucleus might be present at very early embryonic stages. Besides early development, nuclear Olig1 expression in oligodendrocytes was also reported upon remyelination (Arnett et al., 2004). In Olig1-deficient mice, remyelination failed due to impaired differentiation of progenitors, underlining its essential role in oligodendrocyte differentiation (Arnett et al., 2004). In line, we propose Olig1 playing an important role also in Schwann cell differentiation, indicated by its strong differential transcription upon differentiation with forskolin. In the PNS, Olig1 was recently reported to be up-regulated in injured nerves (Arthur-Farraj et al., 2012), suggesting its expression in Schwann cells during denervation and regeneration. Our immunofluorescence analysis on sciatic nerves revealed that Olig1 was localized in regions correlating to nonmyelinating Schwann cells, which is well in line with the decreased mRNA expression level upon forskolin treatment. In addition, Olig1 could be detected in a subset of myelinating Schwann cells, but the immunofluorescent signal was very weak. Furthermore, Olig1 mRNA levels slightly increased during development, reflecting maturation of both myelinating and nonmyelinating Schwann cells. From our data, we introduce Olig1 as a new transcription factor of the Schwann cell lineage, whose expression is negatively regulated by elevation of intracellular cAMP levels by forskolin.

### Regulation of ECM components is forskolin-dependent

Data analysis revealed that a set of differentially expressed transcripts due to forskolin treatment are associated with components of the ECM. Highly significantly increased mRNA expression levels could be identified for the secreted ECM protein spondin 2 and for decorin, a chondroitin sulphate proteoglycan known to be expressed in Schwann cells (Hanemann et al., 1993). These data are in agreement with previous reports showing an up-regulation of decorin during embryonic Schwann cell development (Buchstaller et al., 2004; D’Antonio et al., 2006). Also the proteoglycan thrombomodulin was increased in treated Schwann cells, in line with induced expression of thrombomodulin in endothelial and leukemia cell lines after elevation of intracellular cAMP levels (Ito et al., 1990; Maruyama et al., 1991; Archipoff et al., 1993). To investigate whether differentially expressed gene transcripts might have a common function, they were analyzed in respect to their GO-annotations. Indeed, also this analysis identified the term of ‘extracellular matrix’ for both sets, either transcripts increased or decreased after forskolin treatment. In addition, pathway analysis revealed that the interaction of ECM with its receptors was enriched in differentially expressed genes.

During Schwann cell differentiation and radial sorting *in vivo*, considerable modifications of the plasma membrane occur. The sole axon–glia interaction transforms into a glia–glia interaction on one side, and an axon–glia interaction on the other side of the same membrane elongation. Hence, expressional changes of adhesion molecules and components of the ECM are vital for proper function and polarization *in vivo*. From our data, we conclude that one of the major effects of forskolin is the regulation of the ECM, indicating that changes of the ECM also occur in Schwann cell differentiation *in vitro*.

### Highly significantly differential gene expression induced by elevated cAMP

Schwann cell development and differentiation are also influenced by accurate levels of growth factors. Indeed, Fgf7, also known as keratinocyte growth factor, was identified as one of the strongest forskolin-dependent increased transcripts in primary mouse Schwann cells. This finding is in accordance with studies showing that stimulation with forskolin led to increased Fgf7 expression in other cells, such as dermal papilla cells and primary fibroblasts (Iino et al., 2007; Scott et al., 2012). The strongest induced gene transcript was Sostdc1 (ectodin, wise), an inhibitor of BMP (bone morphogenetic protein) and modulator of the Wnt signaling pathways (Itasaki et al., 2003; Laurikkala et al., 2003). In the CNS, BMP and Wnt signaling pathways inhibit differentiation of oligodendrocyte precursor cells into mature oligodendrocytes (Feigenson et al., 2011), leading to the hypothesis that also in the PNS, forskolin-dependent induction of Sostcd1 expression consequently positively regulates glia cell differentiation. Another significantly induced gene was the endothelin receptor type B. This receptor is coupled to the adenylyl cyclase and was already shown previously to be expressed in immortalized Schwann cells (Wilkins et al., 1997). This increased transcription does not correspond to the forskolin-dependent regulation of its ligand endothelin, which was reduced in treated Schwann cells. However, reduced transcription of endothelin is in line with its proposed function as a negative regulator of the transition from Schwann cell precursors to immature Schwann cells (Brennan et al., 2000). The strongest forskolin-dependent reduction was detected for the transcription of the protocadherin 20 (Pcdh20). This cell adhesion molecule belongs to non-clustered protocadherins, and its expression was analyzed particularly in the CNS (Pribyl et al., 1996). To our knowledge, Pcdh20 expression has not yet been investigated in the PNS. In primary mouse Schwann cells, we detected high expression levels of both transcripts in naive Schwann cells. Upon forskolin treatment, transcription of Pcdh20 was highly significantly reduced, maybe reflecting morphological changes of the cells in conjunction with differentially expressed adhesion molecules. Forskolin treatment resulted also in strongly reduced transcription of the calcium–and calmodulin-dependent phosphodiesterase 1B (Pde1b). As for Pcdh20, gene transcripts of Pde1b were strongly expressed in untreated Schwann cell cultures. This enzyme hydrolyzes the second messengers cAMP and cGMP, consequently regulating their cellular levels (reviewed in Bender and Beavo, 2006; Omori and Kotera, 2007). Hence, elevated intracellular levels of cAMP by forskolin treatment might directly suppress the expression of Pde1b. The third transcript which was strongly reduced in forskolin-treated Schwann cells was the leucine-rich repeat and transmembrane domain-containing protein 1 (Lrtm1). In naive Schwann cells, a strong expression of both transcripts could be detected. The functional role of the Lrtm1 protein is not known, but leucine-rich repeat proteins in general were shown to be involved in cell adhesion and polarization, cytoskeleton dynamics and neural development (reviewed in Kobe and Kajava, 2001), proposing Lrtm1 as a new candidate gene in cell adhesion in Schwann cells. Also for PDGFβ, reduced expression was detected in differentiated Schwann cells, in accordance with reports showing the functional role of PDGF in cell migration and proliferation (De Donatis et al., 2008; Monje et al., 2009; Jiang et al., 2013). Our result of decreased PDGFβ in differentiated Schwann cells is also in agreement with the finding that exogenous PDGF led to suppression of the expression of myelin-related proteins in rat Schwann cells (Ogata et al., 2004).

### Transcripts with reduced expression upon forskolin treatment were associated with the MAPK pathway

Elevation of intracellular cAMP levels went also along with reduced transcription of the transmembrane protein 158 (Tmem158, Ris1). Previously, Tmem158 was shown to be up-regulated in response to activation of the Ras pathway (Barradas et al., 2002; Iglesias et al., 2006; Birch et al., 2008), hence the reduced Tmem158 expression might be a consequence of decreased pathway activity. Since the Ras/Raf/Mek/Erk signaling pathway blocks Schwann cell differentiation (Harrisingh et al., 2004; Ogata et al., 2004), a decreased activity of this pathway is plausible upon differentiation with forskolin. The hypothesis of reduced activity of the MAPK cascade was validated by an analysis of putative pathways, revealing that gene transcripts reduced in treated Schwann cells were often associated with the MAPK pathway. In addition, the MAPK-kinases Mek1/2 could be identified as possible inhibitory target regulators upon forskolin treatment. In line, Rac1 that was proposed as a putative negative target regulator of differentially expressed transcripts in treated Schwann cells, was recently identified as a negative regulator of Schwann cell differentiation by up-regulating cJun and down-regulating Krox20 through the JNK (c-Jun N-terminal kinase) pathway (Shin et al., 2013). Upstream analysis further revealed that three members of the NF-κB pathway are putative target regulators. This is in accordance with the observation, that the activation of NF-κB is vital for peripheral myelination (Nickols et al., 2003). NF-κB was also shown to be phosphorylated and activated by the PKA (Yoon et al., 2008).

### Distinct gene expression between Schwann cells *in vitro* and developing peripheral nerves

PCA demonstrated well-defined clusters of developing peripheral nerves *in vivo*, represented by the time points P0, P4, P7 and P10. Samples of mature sciatic nerves at P60 constituted a separate cluster, indicating distinct gene expression compared with earlier time points. Distinct clusters could also be visualized for treated as well as untreated Schwann cells. Comparison of gene expression between samples derived from primary mouse Schwann cells and samples of developing sciatic nerves revealed significant differences. The differences in gene expression between *in vivo* and *in vitro* might be explained by the fact that there is a constant interaction and reciprocal signaling between axons and Schwann cells *in vivo*, which is absent in primary mouse Schwann cell cultures. Furthermore, the *in vivo* system is more complex, since other cells such as endothelial cells and fibro-blasts are present in the nerve as well. Cultured Schwann cells are more synchronized, in contrast to Schwann cells in peripheral nerves, where an overlap of distinct Schwann cell stages can be observed during early development. These data indicate that caution has to be exercised when comparing primary Schwann cell cultures with *in vivo* analysis, despite the finding that forskolin-treated Schwann cells associate within the same branch of the dendrogram as the nerve samples.

### Conclusion

This work aims to improve the knowledge of intracellular signaling in mouse Schwann cells. Forskolin treatment of primary Schwann cells confirmed the increased transcription of genes involved in Schwann cell differentiation and the reduced transcription of genes known in neural crest cells and Schwann cell precursors. Comprehensive data analysis of a whole-genome microarray further identified a number of differentially regulated transcripts which have not yet been reported in Schwann cells. We identified the expression of both Olig1 protein and mRNA in myelinating and non-myelinating Schwann cells, proposing it as a novel transcription factor in the Schwann cell lineage. From our study, we further conclude that a major effect of forskolin treatment is the regulation of components of the ECM, underlining its importance during Schwann cell differentiation. In addition, transcripts that were reduced upon treatment were often associated with the MAPK and Rac1 signaling pathways, vali-dating previous studies. We provide the whole data set of the microarray study to offer an interactive search tool for genes of interest.

## Online data

Supplementary data

Interactive search tool

Supplementary tables
