# Supplementary material for: Transcriptional regulation induced by cAMP elevation in mouse Schwann cells
Source: ASN Neuro. 2014 Apr 30;6(3):e00142. doi: 10.1042/AN20130031 (PMC4834722; doi:10.1042/AN20130031)
Supplement: Supplementary data [file an006e142add.pdf]

ASN NEURO RESEARCH ARTICLE

ASN NEURO 6(3):art:e00142.doi:10.1042/AN20130031

OPEN ACCESS

Transcriptional regulation induced by cAMP elevation in mouse Schwann cells

Daniela Schmid*, Thomas Zeis* and Nicole Schaeren-Wiemers*[1]

*Neurobiology, Department of Biomedicine, University Hospital Basel, University of Basel, Hebelstrasse 20, CH-4031 Basel, Switzerland

SUPPLEMENTARY DATA

Supplementary Tables S1–S4 and the Interactive Excel file can be found at http://www.asnneuro.org/an/006/an006e142add.htm

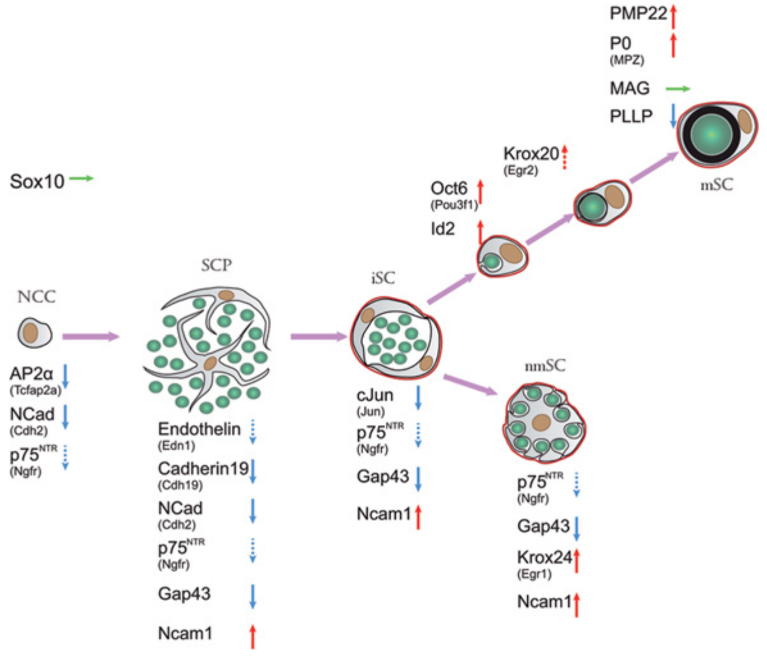

Figure S1 **Schematic illustration of genes implicated in the Schwann cell lineage**
Selected genes of Table 1, which are specific for a distinct Schwann cell stage, were schematically grouped. Genes implicated in neural crest cells, Schwann cell precursors or immature Schwann cells showed reduced mRNA expression levels upon forskolin treatment (blue arrows), whereas increased expression could be detected for myelin-related genes (red arrows). Dashed arrows indicate only slightly differentially expressed transcripts. Red line: basal lamina. NCC: neural crest cell, SCP: Schwann cell precursor, iSC: immature Schwann cell, mSC: myelinating Schwann cell, nmSC: nonmyelinating Schwann cell. Schematic drawing was adapted from Jessen et al. (2005).

[1] To whom correspondence should be addressed (email Nicole.Schaeren-Wiemers@unibas.ch).

© 2014 The Author(s) This is an Open Access article distributed under the terms of the Creative Commons Attribution Licence (CC-BY) (http://creativecommons.org/licenses/by/3.0/) which permits unrestricted use, distribution and reproduction in any medium, provided the original work is properly cited.

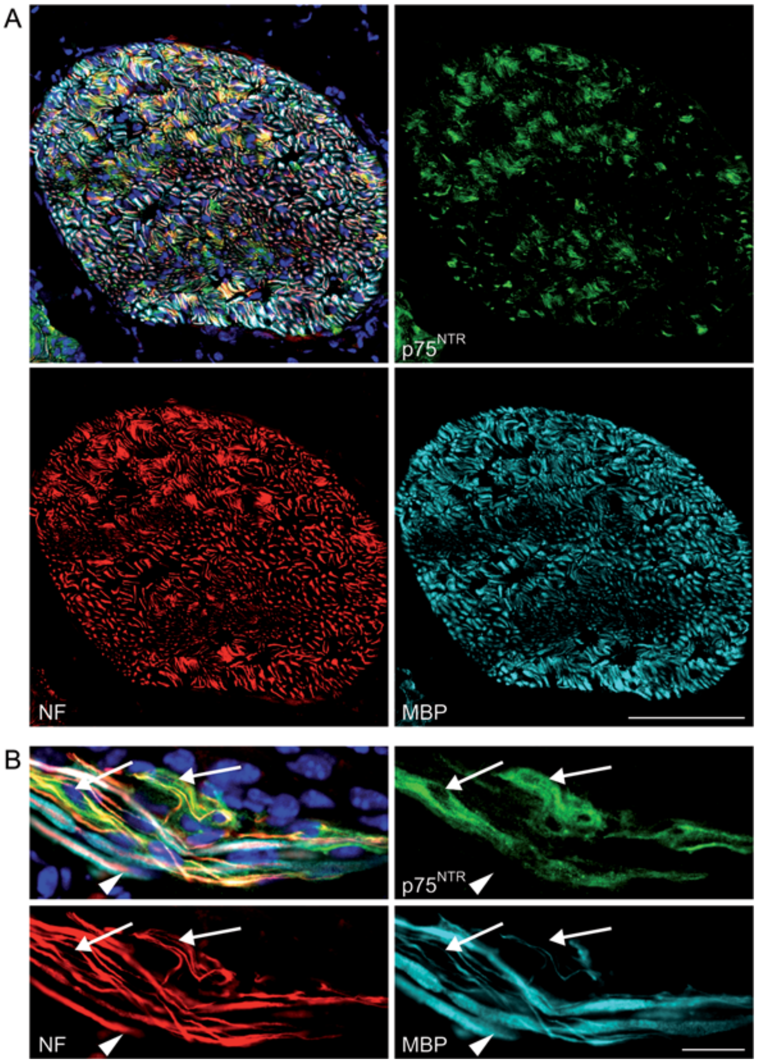

Figure S2 **Localization of p75^{NTR} in nonmyelinating Schwann cells**
Immunofluorescent stainings of transversal (A) and longitudinal (B) tissue sections of sciatic nerves from P7 mice revealed p75^{NTR} immunofluorescence in nonmyelinating Schwann cells (arrows) localized around a bundle of small diameter axons. Myelinating Schwann cells were p75^{NTR}-negative (arrowhead). NF: neurofilament. Bar: A: 100 μm, B: 20 μm.

© 2014 The Author(s) This is an Open Access article distributed under the terms of the Creative Commons Attribution Licence (CC-BY) (http://creativecommons.org/licenses/by/3.0/) which permits unrestricted use, distribution and reproduction in any medium, provided the original work is properly cited.
