# Supplementary material for: Transcriptional regulation induced by cAMP elevation in mouse Schwann cells
Source: ASN Neuro. 2014 Apr 30;6(3):e00142. doi: 10.1042/AN20130031 (PMC4834722; doi:10.1042/AN20130031)
Supplement: Interactive search tool [file an006e142add01.pdf]

Supplementary Table 1: Primer pairs used for qRT-PCR. Analysis of mRNA expression levels by qRT-PCR was performed with the itemized primer pairs. Primer pairs were designed to overlap exon/intron junctions to prevent amplification of genomic DNA.

Gene		Sequence	Gene		Sequence
60s	5':	GGA AGT ACC AGG CAG TGA CAG	Pde1b	5':	GAT GCT GTG CCT TCA GAG GT
	3':	GCA GGC ATG AGG CAA ACA G		3':	GAA CAT CCG CTC CAC GAA GA
Sostdc1	5':	ACT GGA TCG AAA CAG TCG AGT T	Lrtm1	5':	GAA TGA GGG TTT GTG CTG CG
	3':	CCT CCG GCT CCA GTA CTT TG		3':	AAT CAC GGG GGA CCT TTG AC
Plekha4	5':	ACT GCT GAG AAC TAA GGC CC	Pcdh20	5':	CCT GCC GCA TGT TTT TCT GC
	3':	CGC ATG CTT GGT TTC TAC GC		3':	TAC AGA AGC TCG GTA GCA CG
Spon2	5':	ATC GCC AAA GTG ACC TTC GT	Mbp	5':	GGT CCA GGC TTC CTT TGT TTT CTT C
	3':	GCG GTG TCT CTG GAA CTG AA		3':	TGT CCA GCG TGT TCT CCT AAG TCC
Ccl11	5':	AGA GCT CCA CAG CGC TTC TA	Tmem158	5':	GAG GCA CAA ATG AGG TGG GA
	3':	GGA AGT TGG GAT GGA GCC TGG		3':	GGA AAG AGC CGG TTT CCC TT
Egr3	5':	CTC GGT AGC CCA TTA CAA TCA G	Stc2	5':	TGC CCA GGG AAA GTC ATT CA
	3':	ATC GCC GCA GTT GGA ATA AG		3':	AGC ATT CCC TCT GCA ACT GG
Il11	5':	CGC TGG GAC ATT GGG ATC TT	Klhl30	5':	GCG CTA TGA TCC CTA CAC GG
	3':	AGG CGA GAC ATC AAG AGC TG		3':	GAC ACT CCA CGC ATC TGT CA
Egfl8	5':	TCA GGG ACC TCC GAA ATT GTG	Ppp1r1a	5':	GAA GAC CGG ATC CCC AAC TC
	3':	CCT CAC TCT GAG GGC TTC TTC		3':	CGT CTG GAG CTC TTT CAT GGT
Sorcs1	5':	TGA GAT GTG AAG AGC GGG ATT	Atp1b1	5':	CGG TGA CTT TCC CTC CTT GTC
	3':	CTG CCC TGT GCT TTG CAT AC		3':	AAG CTG TAG CTG CAC CAC TCT
Gzmd	5':	ATG TGG TGA AGC CAC ACT CC	Rcan2	5':	GTT GGC TGG AAG CCT ATC
	3':	GGA CAG AGC TGT TTT TGC AGT		3':	CTC TCC TGG TCC TAG TTT GG
Rassf4	5':	GGC ACA ATT CTA GTG CAC GC	Rgs8	5':	TGT CAC TCA GGG TTT CAC CG
	3':	CCA GAG GTC ACC GTT CAG TC		3':	GGT TAA GGA GCT CTG CCT GG
Prss35	5':	CTG GGA CGG GAG GAT ACA GT	Nme7	5':	CAC GGC CCT GAT ACT TTT GC
	3':	GGG GCT GGC AAG ATG GAT AG		3':	CCC AAC ATT CCT TCA CTG ATA GC
Fgf7	5':	GAG AGG CTC AAG TTG CAC GA	Mgp	5':	GCA ACC CTG TGC TAC GAA TC
	3':	CGG TTG CTC CTT GAC TTT TGT		3':	GTA GTC ATC GCA GGC CTC TC
Tgfbr3	5':	AGC TGC CAA AGT GTG TGA CT	Wnt16	5':	CTA TGA GCT GAG TAG CGG CAC
	3':	CAG GGG CTT GGT GAA TGT CT		3':	CAT TCG GTC ATG TTG CCT GC
Kcnn4	5':	GAA GAG AAT CGA CGG TCT G	Klk4	5':	GAA TTC GGA GCC CAG AAG CA
	3':	TCG GTC CTT GGG TAC TTA G		3':	GGA GGC TCC TGT GAC CTC AA
Prss12	5':	TGA CAG CGA TGG ACA TAG GC	Olig1	5':	GCT CCC CAA CAG TGT CTA CC
	3':	TGG CAG GAC CCT TAT AGC CA		3':	ATG GTT TGG CTG GAG AGA GC
Thbd	5':	TAG GGC CCT GGA TCG GTT TA	Pdgfb	5':	TCC GTA GAT GAA GAT GGG GC
	3':	CTG GTG TGG TTA TCG CCA GT		3':	AGC TTT CCA ACT CGA CTC CG
Slc24a3	5':	GGA TCG CAG CCT TCT CCT AC	Acan	5':	GCC AGG GGG AGT TGT ATT CC
	3':	GCC ATA TCC CCC ATC CCT TG		3':	TCA CAC TGC TCA TAG CCT GC
Mpz	5':	CCT CTC AGG TCA CGC TCT ATG	Fhl1	5':	GGG CTG TCA TCA AAG TAG
	3':	GCC CGC TAA CCG CTA TTT C		3':	GCA ATT CTG GAG GGA AAG
Npy5r	5':	AGG GCA TCC CGA GGA CTC TA	Cspg4	5':	TGC TCT ACC GGG TGG TAA GAG G
	3':	TGG AAA GGC AGT GTT CCG AG		3':	GAA GGG CTC AGA GGA CAT CTC G
Ednrb	5':	GCG CAA TGG TCC CAA TAT C	Cav1	5':	CAA CAA CCT CAA CTG CCT ACT C
	3':	TCT GCG AGC AAC TTG TAG G		3':	CCC TAA ACA AAT GGC CGT AAC C
Dcn	5':	TCC CTT ATG ACC CTG ACA ATC C			
	3':	GTC CAA ACC CAG ATC AGA ACA C			

Supplementary Table 2: Differentially expressed genes due to forskolin treatment. A list with differentially expressed genes in treated Schwann cells (20µM forskolin) was compiled with an FDR-adjusted p-value < 0.05. The 25 transcripts with the highest positive or negative fold changes were highlighted in red.

Differentially expressed transcripts due to forskolin		Mean Ratio	Fold Change
Entrez ID	p-value	(20 vs. 0)	(20 vs. 0)
1190002N15Rik	0.000058	0.64	-1.56
1200002N14Rik	0.000123	1.81	1.81
1200009O22Rik	0.000792	1.51	1.51
1700007G11Rik	0.001151	0.62	-1.61
1700021K19Rik	0.000002	0.38	-2.64
1700024G10Rik	0.000123	0.57	-1.75
1700025G04Rik	0.000250	0.66	-1.52
1700029G01Rik	0.000010	1.62	1.62
1810011O10Rik	0.000007	1.71	1.71
2010004A03Rik	0.000020	0.55	-1.82
2210410E06Rik	0.000001	0.58	-1.72
2310022B05Rik	0.000000	0.48	-2.07
2310022M17Rik	0.000140	0.60	-1.66
2310043L02Rik	0.000356	0.56	-1.79
2310043N10Rik	0.000304	1.67	1.67
2410004L22Rik	0.000876	0.60	-1.67
2610524A10Rik	0.000000	0.55	-1.82
2700094K13Rik	0.000059	0.63	-1.59
2810428I15Rik	0.000113	0.60	-1.68
2810439F02Rik	0.000018	1.79	1.79
2810439F02Rik	0.000164	2.17	2.17
3300005D01Rik	0.000069	0.42	-2.39
4632401N01Rik	0.000001	2.01	2.01
4732458O05Rik	0.000590	0.65	-1.55
4732460K03Rik	0.000034	0.43	-2.34
4833412C05Rik	0.000000	0.55	-1.83
4833431D13Rik	0.002122	1.59	1.59
4932416A11Rik	0.000002	1.94	1.94
4933421H10Rik	0.001195	1.62	1.62
5430406J06Rik	0.000006	1.68	1.68
5430435G22Rik	0.000031	0.48	-2.07
5430435G22Rik	0.000001	0.53	-1.90
5530400B01Rik	0.001020	1.53	1.53
5730409K12Rik	0.000002	0.66	-1.50
5730478M09Rik	0.000008	0.61	-1.63
6330403K07Rik	0.000004	2.67	2.67
6330403M23Rik	0.000059	1.58	1.58
9430064K01Rik	0.000273	1.60	1.60
9530064J02	0.000098	0.66	-1.52
9930031P18Rik	0.000422	1.99	1.99
A130010C12Rik	0.000124	1.51	1.51
A130082M07Rik	0.000031	0.62	-1.62
A430103B12Rik	0.000309	1.60	1.60
A530088H08Rik	0.000022	0.53	-1.90
A730085F06Rik	0.000008	1.59	1.59
Aadacl1	0.000185	1.59	1.59
Aatk	0.000216	1.65	1.65
Abcb4	0.000045	0.46	-2.17
Abcc4	0.000040	2.01	2.01
Abr	0.000084	1.83	1.83
Acaa2	0.003101	0.64	-1.55
Acan	0.000000	0.27	-3.72
Acan	0.000000	0.41	-2.43
Acot1	0.000001	1.99	1.99
Acta2	0.000053	0.64	-1.57
Acta2	0.000068	0.65	-1.55
Acta2	0.000005	0.60	-1.65
Actg2	0.000016	0.61	-1.64
Acvr1	0.000391	0.66	-1.51
Adamts1	0.000306	0.61	-1.63
Adamts12	0.000001	0.52	-1.93
Adamts15	0.000116	1.56	1.56
Adamtsl5	0.000251	0.61	-1.65
Add3	0.000000	2.24	2.24
Add3	0.000000	2.00	2.00
Aebp1	0.000212	1.51	1.51
Agrn	0.000056	0.60	-1.66
Alcam	0.000004	2.07	2.07
Aldh1a3	0.000514	0.53	-1.89
Aldh1a3	0.000066	0.37	-2.71
Ankrd39	0.000578	0.64	-1.57
Anp32e	0.000013	0.63	-1.59
Anxa1	0.000020	0.55	-1.81

Differentially expressed transcripts due to forskolin		Mean Ratio	Fold Change
Entrez ID	p-value	(20 vs. 0)	(20 vs. 0)
Aoc3	0.004084	1.65	1.65
Apbb2	0.000860	0.58	-1.74
Appl2	0.000006	1.83	1.83
Atf3	0.000309	0.30	-3.32
Atp1a2	0.000001	0.65	-1.53
Atp1b1	0.000028	0.20	-4.97
Axud1	0.000509	0.63	-1.59
Baalc	0.000001	2.94	2.94
Baalc	0.000000	2.67	2.67
BC020002	0.000003	0.58	-1.74
BC020108	0.000497	1.92	1.92
BC050210	0.000374	0.66	-1.51
Bcl2l11	0.000002	2.04	2.04
Bcl3	0.002488	1.65	1.65
Bcl7a	0.000049	1.58	1.58
Bmf	0.000011	2.66	2.66
Bmp4	0.002119	1.54	1.54
Bok	0.000087	0.61	-1.64
Btbd11	0.000002	0.64	-1.57
C1qtnf1	0.000020	1.65	1.65
C1qtnf3	0.000535	0.58	-1.73
C1qtnf3	0.000110	0.62	-1.60
Cacna1h	0.000931	1.69	1.69
Calb2	0.000450	0.42	-2.39
Camk2n1	0.000029	2.10	2.10
Camk2n1	0.000005	2.11	2.11
Capn5	0.000282	1.69	1.69
Cav1	0.000000	0.29	-3.49
Ccdc80	0.000057	0.63	-1.58
Ccl11	0.000186	6.20	6.20
Ccl11	0.000004	10.68	10.68
Ccl7	0.003240	2.16	2.16
Ccl7	0.000333	1.92	1.92
Ccrn4l	0.000008	0.58	-1.73
Cd109	0.000001	0.42	-2.37
Cd109	0.000000	0.32	-3.14
Cd200	0.000000	0.51	-1.97
Cd200	0.000032	0.62	-1.61
Cd80	0.001090	0.60	-1.67
Cda	0.003023	1.51	1.51
Cdca3	0.003955	0.65	-1.53
Cdh10	0.000047	0.56	-1.80
Cdh19	0.002132	0.62	-1.62
Cdh19	0.000342	0.59	-1.69
Cdh5	0.003800	1.50	1.50
Cdk5rap2	0.000423	0.63	-1.58
Cdkn1a	0.001536	0.59	-1.71
Cdkn1c	0.003230	1.80	1.80
Ceacam2	0.001093	1.58	1.58
Cebpb	0.000479	2.04	2.04
Ch25h	0.000010	0.54	-1.87
Chst2	0.002840	0.65	-1.53
Ckb	0.000006	0.65	-1.55
Clasp1	0.000006	1.53	1.53
Clca4	0.001403	1.50	1.50
Clcf1	0.000005	1.69	1.69
Clcf1	0.000107	1.54	1.54
Clcf1	0.000077	1.58	1.58
Clmn	0.000030	1.62	1.62
Cnksr2	0.000001	0.51	-1.96
Cnn2	0.003388	0.67	-1.50
Col14a1	0.000005	2.73	2.73
Col14a1	0.000000	2.63	2.63
Col15a1	0.000009	0.48	-2.10
Col28a1	0.000051	0.58	-1.72
Col4a5	0.000017	0.63	-1.60
Col5a2	0.003559	0.66	-1.51
Col8a1	0.001428	0.63	-1.59
Coro2b	0.000026	1.77	1.77
Cpne8	0.001910	0.61	-1.64
Crabp2	0.000175	2.09	2.09
Creb5	0.000039	0.52	-1.92
Crhbp	0.000006	1.83	1.83

Supplementary Table 2: continued

Differentially expressed transcripts due to forskolin

Entrez ID	p-value	Mean Ratio (20 vs. 0)	Fold Change (20 vs. 0)
Crispld2	0.000948	1.61	1.61
Crlf1	0.000001	1.53	1.53
Cryab	0.000033	0.55	-1.83
Csmd2	0.000700	1.63	1.63
Cspg4	0.000000	0.28	-3.52
Csrp3	0.000332	0.64	-1.56
Cthrc1	0.000000	2.39	2.39
Ctsb	0.002984	1.53	1.53
Cx3cl1	0.003110	1.76	1.76
Cxcl1	0.002912	2.01	2.01
Cxcl12	0.003575	1.91	1.91
Cxcl12	0.001442	1.81	1.81
Cxcl14	0.000115	2.57	2.57
Cxcl14	0.000860	1.72	1.72
Cyp2d22	0.000081	1.66	1.66
D030063E12	0.000000	1.86	1.86
D030072B18Rik	0.000001	0.66	-1.51
D0H4S114	0.000004	1.66	1.66
D12Ertd553e	0.000619	1.68	1.68
D16Ertd472e	0.000045	1.55	1.55
D1Ertd471e	0.002910	0.60	-1.67
D330020A13Rik	0.000001	1.85	1.85
D330027H18Rik	0.000000	0.60	-1.66
D630040I23Rik	0.000030	0.60	-1.67
Dap	0.000018	0.66	-1.51
Dbndd2	0.000000	0.48	-2.06
Dclk1	0.000575	1.73	1.73
Dcn	0.002973	2.90	2.90
Dcn	0.000668	3.16	3.16
Ddit4	0.000000	2.27	2.27
Ddit4l	0.003681	1.50	1.50
Depdc6	0.000878	1.59	1.59
Des	0.000003	0.50	-1.99
Dhh	0.000000	1.80	1.80
Dhx32	0.000032	0.57	-1.75
Diras1	0.000001	0.41	-2.47
Dlk1	0.000515	1.69	1.69
Dlk1	0.000030	2.10	2.10
Dm15	0.000049	0.58	-1.73
Dok5	0.000002	0.59	-1.70
Dok5	0.000147	0.64	-1.55
Dst	0.000612	1.55	1.55
Dstn	0.000018	0.67	-1.50
Dtna	0.000000	0.40	-2.51
Dtna	0.000082	0.60	-1.67
Dusp4	0.000370	0.66	-1.52
Dusp4	0.000914	0.61	-1.64
Dusp8	0.000026	1.71	1.71
Dysf	0.000000	0.55	-1.82
E230013M07Rik	0.000000	5.49	5.49
E330035H20Rik	0.000004	1.71	1.71
Ednrb	0.000001	3.25	3.25
Ednrb	0.000000	2.32	2.32
Ednrb	0.000000	2.05	2.05
EG574403	0.002279	1.53	1.53
Egfl8	0.000068	6.19	6.19
Egfl8	0.000104	5.04	5.04
Egr3	0.000001	7.26	7.26
Ehd4	0.000029	0.62	-1.60
Emb	0.002300	2.50	2.50
En1	0.000490	0.52	-1.93
Enc1	0.000343	0.35	-2.88
Endod1	0.000006	0.48	-2.09
Entpd2	0.004328	1.92	1.92
Epb4.1l4b	0.000001	2.62	2.62
Epb4.1l4b	0.000001	2.66	2.66
Errfi1	0.000249	2.87	2.87
Ets1	0.000000	0.58	-1.72
Fabp3	0.000109	0.57	-1.75
Fam132b	0.000808	0.62	-1.61
Fam134b	0.000030	2.04	2.04
Fam171b	0.002227	0.42	-2.40
Farp2	0.000002	1.85	1.85

Differentially expressed transcripts due to forskolin

Entrez ID	p-value	Mean Ratio (20 vs. 0)	Fold Change (20 vs. 0)
Fgf7	0.000437	3.78	3.78
Fgfrl1	0.000243	1.97	1.97
Fhl1	0.000000	0.27	-3.64
Fhl1	0.000000	0.32	-3.13
Fjx1	0.000168	1.97	1.97
Fjx1	0.000243	1.65	1.65
Fkhl18	0.000072	0.54	-1.86
Flt1	0.000003	0.52	-1.94
Fmn2	0.000246	1.57	1.57
Fmnl3	0.000000	0.59	-1.70
Fmnl3	0.000001	0.56	-1.80
Fmod	0.001786	2.03	2.03
Foxi2	0.000000	1.88	1.88
Foxo1	0.001664	0.55	-1.81
Frmd4a	0.000512	1.63	1.63
Fxyd3	0.001011	2.34	2.34
Fxyd5	0.000003	0.61	-1.65
Fxyd5	0.000181	0.64	-1.56
Fxyd5	0.000149	0.64	-1.57
Fyb	0.000083	0.56	-1.78
Fzd2	0.000048	1.52	1.52
Fzd6	0.000004	1.80	1.80
Fzd6	0.000000	2.17	2.17
Gab1	0.000001	0.42	-2.41
Gabrb3	0.000818	0.53	-1.89
Gabrb3	0.000046	0.59	-1.70
Gadd45b	0.000003	0.54	-1.87
Galnt2	0.000000	1.61	1.61
Galnt2	0.000003	1.74	1.74
Galnt2	0.000020	1.84	1.84
Galntl4	0.001064	0.53	-1.88
Gap43	0.000036	0.50	-2.00
Gas2l3	0.000054	1.68	1.68
Gas2l3	0.001408	1.59	1.59
Gdf1	0.000021	1.73	1.73
Gdnf	0.002929	0.65	-1.54
Gdpd5	0.000309	1.66	1.66
Gfap	0.004051	1.52	1.52
Gja1	0.002280	1.80	1.80
Glis1	0.000486	1.82	1.82
Gng2	0.001858	1.63	1.63
Gng2	0.000472	1.74	1.74
Gnptab	0.000001	1.57	1.57
Gp38	0.000927	1.65	1.65
Gpbar1	0.000420	0.63	-1.58
Gpr137b	0.000095	1.60	1.60
Gpr153	0.000252	1.52	1.52
Gpr176	0.000000	0.41	-2.43
Gpr23	0.000011	1.58	1.58
Gse1	0.000033	1.59	1.59
Gsn	0.001129	0.62	-1.61
Gsta4	0.000677	1.67	1.67
Gstm1	0.000002	1.63	1.63
Gucy1a3	0.002333	0.61	-1.65
Gzmd	0.002338	1.61	1.61
Gzmd	0.002419	4.95	4.95
Gzme	0.003553	3.09	3.09
Gzme	0.003961	2.22	2.22
Hdac4	0.002296	1.54	1.54
Hdac5	0.000754	0.65	-1.53
Hdac5	0.000002	0.60	-1.67
Hebp1	0.000440	0.63	-1.59
Heyl	0.000240	0.57	-1.74
Hist1h2af	0.000275	0.66	-1.52
Hist1h2ah	0.004376	0.63	-1.60
Hmga1	0.000010	0.53	-1.89
Hs2st1	0.000000	0.66	-1.51
Hs3st3a1	0.000307	0.55	-1.80
Hsd11b1	0.000535	1.60	1.60
Hsd17b11	0.000239	1.59	1.59
Hspa1l	0.000110	0.61	-1.65
Hspb1	0.000062	0.38	-2.66
Hspb2	0.000037	0.53	-1.88
Hspb3	0.000005	0.50	-2.00
Htra3	0.000998	0.63	-1.59

Supplementary Table 2: continued

Differentially expressed transcripts due to forskolin

Entrez ID	p-value	Mean Ratio (20 vs. 0)	Fold Change (20 vs. 0)
Id1	0.002180	1.50	1.50
Id2	0.000000	2.12	2.12
Idb2	0.000000	3.03	3.03
Ifit2	0.002078	0.66	-1.51
Igf1	0.000049	0.63	-1.60
Igf1	0.000001	0.53	-1.89
Igfbp3	0.000001	0.40	-2.47
Igfbp3	0.000227	0.50	-2.02
Igfbp5	0.000000	1.85	1.85
Igfbp5	0.000001	2.09	2.09
Igfbp5	0.000000	2.14	2.14
Igsf11	0.000003	2.77	2.77
Igsf11	0.000007	2.77	2.77
Igsf4a	0.000000	0.46	-2.16
Ikbkg	0.000001	1.63	1.63
Il11	0.000000	7.24	7.24
Il33	0.003905	1.75	1.75
Il6	0.002469	1.51	1.51
Inpp5a	0.000009	0.55	-1.83
Inpp5a	0.000004	0.53	-1.89
Inpp5f	0.002289	1.59	1.59
Inppl1	0.000558	0.65	-1.54
Iqgap2	0.000585	2.17	2.17
Irf6	0.000001	0.38	-2.61
Irgm1	0.004311	0.66	-1.51
Itga1	0.000000	0.49	-2.05
Itga5	0.000262	0.64	-1.56
Itgb4	0.000036	1.63	1.63
Itgb4	0.000177	1.68	1.68
Itgb5	0.000096	0.54	-1.86
Itgb8	0.000002	1.59	1.59
Itpk1	0.001470	0.63	-1.59
Ivl	0.000291	0.56	-1.79
Jun	0.000020	0.62	-1.61
Kcna6	0.000028	1.60	1.60
Kcna6	0.000011	1.62	1.62
Kcnab1	0.000363	0.65	-1.54
Kcnab1	0.003562	0.67	-1.50
Kcnh2	0.000017	2.08	2.08
Kcnh2	0.000028	1.77	1.77
Kcnj12	0.000169	0.64	-1.55
Kcnk13	0.000001	0.40	-2.49
Kcnk5	0.000000	2.08	2.08
Kcnk5	0.000000	2.12	2.12
Kcnn4	0.000000	3.62	3.62
Kif21a	0.000013	1.59	1.59
Kif26a	0.000000	1.51	1.51
Kitl	0.000289	1.76	1.76
Klf13	0.000042	1.75	1.75
Klhl26	0.000002	1.70	1.70
Klhl30	0.000000	0.14	-7.04
Klk4	0.000008	0.25	-3.95
Klk8	0.000001	0.33	-3.04
Krt23	0.001666	0.64	-1.57
Lama1	0.002195	1.75	1.75
Lbh	0.000000	0.35	-2.88
Lbh	0.000000	0.34	-2.98
Leprel1	0.000001	0.52	-1.93
Leprel1	0.000000	0.34	-2.94
Lgi4	0.000215	1.87	1.87
Lgmn	0.000000	0.52	-1.94
Lgr6	0.000055	0.66	-1.50
Lhfp	0.000068	1.68	1.68
Lhfp	0.000288	1.76	1.76
Lhfp	0.000142	1.52	1.52
Lims2	0.000000	0.42	-2.38
Lims2	0.000000	0.45	-2.23
Lims2	0.000000	0.49	-2.05
Lip1	0.000001	0.53	-1.87
Lip1	0.000084	0.61	-1.64
Liph	0.000176	1.82	1.82
Liph	0.000193	2.34	2.34
LOC100044177	0.000006	3.09	3.09

Differentially expressed transcripts due to forskolin

Entrez ID	p-value	Mean Ratio (20 vs. 0)	Fold Change (20 vs. 0)
LOC100045228	0.000279	2.20	2.20
LOC100047214	0.000075	1.63	1.63
LOC100047268	0.000292	2.02	2.02
LOC100047583	0.000444	0.35	-2.82
LOC100047606	0.000006	1.82	1.82
LOC100047619	0.000125	0.63	-1.58
LOC100047659	0.000003	3.07	3.07
LOC100047808	0.000013	0.41	-2.47
LOC100048721	0.000468	1.52	1.52
LOC218877	0.000013	1.72	1.72
LOC234081	0.000026	0.53	-1.88
LOC240672	0.000010	0.40	-2.50
LOC638935	0.000007	0.52	-1.92
LOC666036	0.000000	0.54	-1.85
Loxl4	0.000133	0.55	-1.82
Lpcat2	0.001179	0.58	-1.74
Lrrc17	0.002276	1.71	1.71
Lrrn3	0.000000	0.42	-2.39
Lrrtm2	0.000941	1.92	1.92
Lrtm1	0.000010	0.09	-11.60
Lrtm1	0.000018	0.09	-10.92
Lum	0.001457	1.98	1.98
Lypd1	0.000378	0.64	-1.57
Mapk9	0.000262	0.65	-1.53
Matn2	0.000001	1.62	1.62
Mbp	0.000000	0.10	-9.98
Mbp	0.000016	0.40	-2.51
Mcf2l	0.000045	0.55	-1.81
Mdga2	0.000225	0.65	-1.53
Mdh1	0.000175	0.59	-1.70
Me2	0.000729	0.66	-1.51
Mef2c	0.000068	0.46	-2.19
Mef2c	0.000000	0.33	-3.03
Mef2c	0.000000	0.36	-2.81
Meox1	0.000124	0.67	-1.50
Mertk	0.002382	1.64	1.64
Mfap2	0.000007	1.55	1.55
Mfap2	0.000203	1.69	1.69
Mgll	0.000063	0.57	-1.76
Mgll	0.000002	0.46	-2.18
Mgll	0.000180	0.53	-1.88
Mgp	0.000448	0.25	-4.01
Mgst1	0.000900	1.88	1.88
Mgst1	0.000695	2.66	2.66
Mid1ip1	0.000558	0.66	-1.52
Mid2	0.000001	0.40	-2.49
Mmd2	0.001290	1.50	1.50
Mmp15	0.000371	1.90	1.90
Mmp17	0.000003	0.41	-2.45
Moxd1	0.000020	0.58	-1.73
Moxd1	0.000012	0.59	-1.70
Mpz	0.000072	3.41	3.41
Myl9	0.000527	0.64	-1.57
Myom1	0.000651	0.66	-1.53
Ncald	0.003406	1.58	1.58
Ncam1	0.000030	2.20	2.20
Ncam1	0.002629	1.52	1.52
Ndrg1	0.000000	2.21	2.21
Ndrl	0.000000	2.12	2.12
Nedd4l	0.000017	0.65	-1.53
Nek1	0.001319	1.63	1.63
Nid1	0.000002	1.77	1.77
Nid1	0.000050	1.88	1.88
Nme7	0.000042	0.55	-1.80
Nme7	0.000002	0.24	-4.12
Nme7	0.000011	0.29	-3.42
Nnat	0.000008	2.15	2.15
Nppb	0.000184	0.54	-1.84
Npr3	0.002365	2.86	2.86
Npr3	0.000730	1.59	1.59
Npr3	0.001797	1.84	1.84
Npy1r	0.000012	2.37	2.37
Npy5r	0.000007	2.79	2.79
Npy5r	0.000000	3.31	3.31
Nr2f2	0.000111	1.66	1.66

Supplementary Table 2: continued

Differentially expressed transcripts due to forskolin Entrez ID	p-value	Mean Ratio (20 vs. 0)	Fold Change (20 vs. 0)
Nrbp2	0.000756	1.68	1.68
Nupr1	0.000923	0.57	-1.76
Olfml3	0.000735	1.86	1.86
Olig1	0.000857	0.25	-3.94
Osbpl3	0.000140	1.50	1.50
OTTMUSG00000002196	0.000113	0.43	-2.31
P2rx5	0.000000	0.36	-2.80
P2ry1	0.000036	0.60	-1.67
Pacsin2	0.000118	0.66	-1.52
Padi2	0.000025	0.62	-1.62
Parp14	0.000000	2.04	2.04
Pbk	0.001506	0.58	-1.73
Pcdh17	0.001548	0.54	-1.87
Pcdh20	0.000000	0.07	-14.02
Pcdh20	0.000000	0.10	-10.07
Pdcd4	0.000169	1.53	1.53
Pde10a	0.000072	1.52	1.52
Pde1a	0.002561	1.71	1.71
Pde1a	0.002365	2.00	2.00
Pde1b	0.000000	0.09	-11.30
Pde1b	0.000000	0.08	-12.05
Pde4b	0.000000	2.54	2.54
Pde4d	0.000069	1.71	1.71
Pde7b	0.000053	1.84	1.84
Pdgfa	0.000747	0.64	-1.57
Pdgfb	0.000314	0.26	-3.84
Pea15	0.000001	0.53	-1.89
Pea15a	0.000191	0.57	-1.74
Pgf	0.000002	0.44	-2.27
Phgdh	0.000025	0.53	-1.90
Pik3r1	0.000294	1.69	1.69
Pitpnc1	0.003041	1.62	1.62
Pkig	0.000001	1.71	1.71
Pla2g7	0.000083	1.93	1.93
Plau	0.000221	2.41	2.41
Pld3	0.000022	1.71	1.71
Plekha4	0.000000	4.15	4.15
Plekha4	0.000000	17.53	17.53
Plekhb1	0.000000	1.67	1.67
Pllp	0.001800	0.64	-1.57
Plxna2	0.000214	0.58	-1.71
Pmaip1	0.002204	0.65	-1.55
Pmepa1	0.000323	1.62	1.62
Pmepa1	0.000526	1.56	1.56
Pmp22	0.003731	1.52	1.52
Ppap2a	0.000226	0.47	-2.11
Ppap2a	0.000001	0.47	-2.11
Ppap2a	0.000000	0.38	-2.66
Ppap2b	0.001931	2.76	2.76
Ppap2b	0.000091	2.81	2.81
Ppbp	0.001434	1.71	1.71
Ppbp	0.001813	1.94	1.94
Ppm2c	0.000696	0.65	-1.53
Ppp1r1a	0.000002	0.17	-5.83
Ppp3ca	0.000000	2.16	2.16
Ppp3ca	0.000026	2.23	2.23
Prelp	0.000006	1.66	1.66
Prkg1	0.000041	0.34	-2.90
Prkg1	0.000000	0.40	-2.51
Prl2c2	0.001734	1.57	1.57
Pros1	0.001808	1.64	1.64
Prss12	0.000004	3.61	3.61
Prss12	0.000005	3.16	3.16
Prss23	0.000005	0.53	-1.89
Prss35	0.000339	4.18	4.18
Psmd8	0.000000	0.61	-1.64
Ptgis	0.001424	0.66	-1.51
Ptk2	0.000012	0.64	-1.56
Ptn	0.000005	2.82	2.82
Ptpla	0.000513	0.64	-1.55
Ptprn	0.000128	1.51	1.51
Ptprt	0.000007	0.64	-1.56
Ptpru	0.000005	1.62	1.62

Differentially expressed transcripts due to forskolin Entrez ID	p-value	Mean Ratio (20 vs. 0)	Fold Change (20 vs. 0)
Ptpru	0.000054	1.88	1.88
Ptprz1	0.000096	0.30	-3.32
Ramp3	0.000009	2.31	2.31
Ramp3	0.000019	2.88	2.88
Rap2a	0.000000	2.33	2.33
Rasl12	0.000656	0.60	-1.66
Rassf4	0.002989	1.54	1.54
Rassf4	0.004456	4.34	4.34
Rbms3	0.000031	1.81	1.81
Rbmx	0.000060	0.65	-1.54
Rbpms2	0.000045	0.66	-1.52
Rcan2	0.000008	0.21	-4.87
Rcan2	0.000000	0.35	-2.85
Rcsd1	0.001466	0.66	-1.52
Rdh5	0.000477	1.66	1.66
Reln	0.000030	2.73	2.73
Rftn1	0.000688	0.60	-1.66
Rgs16	0.000001	0.33	-3.01
Rgs3	0.000000	0.58	-1.72
Rgs8	0.000010	0.22	-4.61
Rhoc	0.000149	0.67	-1.50
Rin1	0.000002	0.63	-1.60
Rnf125	0.001748	1.68	1.68
Robo2	0.000845	1.62	1.62
Rorb	0.000038	1.62	1.62
Rrm2	0.000226	0.64	-1.56
Rxfp3	0.000103	0.60	-1.67
Satb1	0.000000	0.32	-3.16
Scd1	0.000039	1.61	1.61
scl000981.1_40	0.003147	1.56	1.56
Sema3b	0.000002	2.00	2.00
Sema3b	0.000436	1.69	1.69
Sema6b	0.002104	1.83	1.83
Sema6b	0.000987	1.71	1.71
39692	0.000066	0.66	-1.51
Sfrp1	0.003690	1.96	1.96
Sfrp1	0.000487	1.88	1.88
Sgk1	0.002665	1.89	1.89
Sgsm3	0.000001	1.56	1.56
Shh	0.000420	2.49	2.49
Slamf9	0.000341	0.61	-1.63
Slc16a11	0.000009	1.52	1.52
Slc17a8	0.000000	1.89	1.89
Slc24a3	0.000037	3.53	3.53
Slit2	0.000181	1.80	1.80
Slit3	0.000000	1.83	1.83
Smad3	0.000166	1.57	1.57
Snta1	0.000001	0.49	-2.03
Snx10	0.000069	1.57	1.57
Snx16	0.000374	2.69	2.69
Snx7	0.001131	0.62	-1.61
Socs3	0.003840	1.85	1.85
Sod3	0.000072	2.31	2.31
Sorcs1	0.000000	2.55	2.55
Sorcs1	0.000000	5.68	5.68
Sorcs1	0.000002	1.55	1.55
Sostdc1	0.000002	35.54	35.54
Spon2	0.001100	14.12	14.12
Spsb4	0.000001	0.40	-2.48
Srpx2	0.002500	0.63	-1.58
Ssbp2	0.000009	1.52	1.52
St3gal5	0.000085	0.55	-1.81
Stc1	0.000171	1.70	1.70
Stc2	0.000039	0.11	-8.82
Stmn4	0.000256	0.60	-1.67
Stmn4	0.000060	0.61	-1.65
Sv2b	0.001656	0.55	-1.82
Svep1	0.001823	2.53	2.53
Syn1	0.000052	1.71	1.71
Syn1	0.001779	1.58	1.58
Synpo	0.000129	1.53	1.53
Tbc1d7	0.001003	1.77	1.77
Tcap	0.000035	0.33	-3.02
Tcap	0.000752	0.55	-1.81
Tcf3	0.000008	1.51	1.51

Supplementary Table 2: continued

Differentially expressed transcripts due to forskolin<br>Entrez ID	p-value	Mean Ratio<br>(20 vs. 0)	Fold Change<br>(20 vs. 0)
Tctn3	0.000012	1.54	1.54
Tctn3	0.000000	2.05	2.05
Tgfbr3	0.000000	3.72	3.72
Tgfbr3	0.002110	1.50	1.50
Tgm2	0.000025	0.48	-2.10
Thbd	0.000091	3.59	3.59
Thbs1	0.000005	0.50	-1.99
Thsd4	0.000024	0.47	-2.11
Tia1	0.000302	1.51	1.51
Tmem100	0.000338	1.89	1.89
Tmem158	0.000165	0.11	-8.85
Tmem45a	0.000134	1.58	1.58
Tmod2	0.000005	1.80	1.80
Tnc	0.001404	0.58	-1.71
Tnc	0.000002	0.57	-1.76
Tnik	0.000012	0.53	-1.90
Tnik	0.000147	0.48	-2.07
Tnk2	0.004450	1.50	1.50
Tns3	0.000004	0.67	-1.50
Trib2	0.000014	0.60	-1.68
Trim2	0.000595	1.51	1.51
Trim47	0.000206	0.54	-1.85
Trp53inp2	0.000030	1.59	1.59
Tsc22d4	0.003080	1.87	1.87
Ttll5	0.000044	1.78	1.78
Ttll5	0.000165	1.60	1.60
Twist2	0.003283	1.58	1.58
Ube2e2	0.000508	0.57	-1.77
Uck2	0.000010	0.62	-1.61
Uck2	0.000436	0.61	-1.63
Ugp2	0.000001	0.36	-2.80
Ugt1a10	0.000147	2.43	2.43
Ugt1a10	0.000002	2.05	2.05
Ugt1a10	0.000090	2.20	2.20
Ugt1a6a	0.000643	1.64	1.64
Ugt1a6a	0.000034	1.91	1.91
Ugt1a6b	0.000006	2.45	2.45
Usp6nl	0.000011	1.65	1.65
Vamp5	0.000475	0.60	-1.68
Vcan	0.001572	1.97	1.97
Vcan	0.001250	2.28	2.28
Vcan	0.001253	2.09	2.09
Vdr	0.002828	1.91	1.91
Vsnl1	0.002117	0.64	-1.57
Vstm2b	0.000515	1.66	1.66
Wnt10b	0.000000	0.48	-2.10
Wnt16	0.002631	0.39	-2.59
Wnt16	0.000012	0.25	-4.01
Wnt16	0.000001	0.28	-3.56
Xdh	0.001479	1.62	1.62
Ybx3	0.001143	1.83	1.83
Zfp185	0.001445	0.59	-1.70
Zfp219	0.000019	1.50	1.50
Zfp704	0.000402	1.52	1.52

Supplementary Table 3: Differential gene expression upon forskolin treatment. Raw data of the Figure 1. The mRNA expression levels of selected genes was investigated by qRT-PCR. Data were normalized to the expression of 60s. The columns represent the mean value of twelve experimental samples, and the error bars indicate the SEM. n.d.: not detected

		0 µM Forskolin												20 µM Forskolin														pvalue
		1	2	3	4	5	6	7	8	9	10	11	12	1	2	3	4	5	6	7	8	9	10	11	12	0 µM	20 µM	
A	Sostdc1	0.62	0.56	1.01	1.13	1.13	n.d.	1.45	0.79	0.67	1.26	1.15	1.24	8.42	11.49	10.37	10.24	8.77	16.00	19.51	5.65	6.02	9.25	11.51	12.22	1.00	10.79	0.0000028
	Plekha4	0.25	n.d.	0.44	0.36	0.68	0.42	0.58	0.22	5.67	0.41	0.97	n.d.	116.60	91.81	41.61	68.56	68.56	87.04	42.73	28.79	37.80	21.32	23.56	66.06	1.00	57.87	0.0000416
	Spon2	1.37	1.33	1.07	1.88	1.15	1.09	1.47	0.58	0.71	0.48	0.28	0.59	6.60	7.49	8.31	10.47	6.94	7.07	10.85	3.92	3.41	3.74	4.39	2.09	1.00	6.27	0.0000384
	Ccl11	1.61	0.97	0.83	0.98	0.92	1.26	1.87	1.13	0.71	0.69	0.57	0.43	23.33	18.06	39.12	25.07	25.92	30.51	27.63	13.10	14.36	14.55	14.98	18.68	1.00	22.11	0.0000016
	Egr3	0.48	0.53	0.53	0.94	0.59	0.67	1.11	2.15	1.47	1.03	1.33	1.19	21.55	22.41	10.59	12.71	8.41	14.10	13.26	7.80	6.75	6.68	8.03	8.44	1.00	11.73	0.0000257
	Il11	2.30	2.94	0.72	1.30	1.29	0.81	0.86	0.32	0.39	0.36	0.38	0.33	28.29	26.59	12.02	13.05	10.11	13.28	13.05	9.08	8.11	8.79	9.24	11.26	1.00	13.57	0.0000428
	Egfl8	0.86	0.81	0.95	1.34	1.04	0.99	1.21	1.04	0.90	0.97	0.81	1.07	6.08	8.14	5.39	5.46	5.20	6.35	5.43	2.66	2.85	3.19	3.29	4.59	1.00	4.89	0.0000049
	Sorcs1	1.48	1.40	1.14	1.40	1.21	0.89	0.97	0.93	0.58	0.67	0.58	0.75	8.30	6.67	5.37	5.08	4.39	4.19	3.62	2.47	2.76	2.55	2.78	3.86	1.00	4.34	0.0000398
	Gzmd	0.29	0.53	1.99	1.98	1.45	1.44	2.23	0.50	0.70	0.44	0.35	0.10	15.35	16.63	31.22	33.38	37.01	43.68	39.01	31.01	22.35	18.70	22.78	25.48	1.00	28.05	0.0000006
	Rassf4	0.55	0.33	1.01	1.29	1.08	1.39	1.85	0.82	0.77	0.95	1.13	0.84	1.88	1.69	2.41	2.74	2.04	5.97	5.31	1.49	1.83	1.95	2.83	2.93	1.00	2.76	0.0013174
	Prss35	1.10	0.76	0.97	1.22	1.06	1.38	1.42	0.77	0.76	0.84	0.81	0.92	1.52	1.24	1.53	1.77	1.55	2.27	2.24	1.02	1.05	1.38	1.19	1.81	1.00	1.55	0.0009430
	Fgf7	0.59	0.61	0.60	0.96	0.76	1.33	1.49	1.35	1.35	0.83	1.11	1.01	5.25	4.42	5.90	6.65	4.96	6.84	7.52	6.42	5.41	5.13	5.84	7.99	1.00	6.03	0.0000000
	Tgfbr3	1.26	0.94	1.05	1.31	1.10	0.20	1.14	1.06	0.88	1.17	0.98	0.91	7.37	7.61	5.77	5.83	4.79	5.88	4.95	3.06	3.02	3.07	3.81	4.41	1.00	4.96	0.0000023
	Kcnn4	1.38	1.41	0.96	1.41	1.22	0.90	0.96	0.88	0.74	0.57	0.71	0.86	7.92	6.56	4.87	5.35	3.88	3.83	3.13	2.12	2.03	2.23	2.79	3.42	1.00	4.01	0.0001418
	Prss12	1.10	1.41	1.16	1.62	1.19	0.95	1.21	0.65	0.66	0.73	0.63	0.70	6.67	7.23	7.15	6.31	4.71	7.06	16.43	3.45	3.08	3.37	4.41	4.98	1.00	6.24	0.0003451
	Thbd	0.82	0.60	1.10	1.58	1.01	1.22	1.75	0.78	0.87	0.76	0.75	0.77	4.35	5.16	6.26	8.57	6.47	9.32	9.14	5.57	4.68	5.96	6.02	7.42	1.00	6.58	0.0000001
	Slc24a3	0.47	0.54	0.70	1.36	1.12	0.93	1.45	1.11	1.24	0.99	1.17	0.92	5.76	7.63	3.55	4.87	3.18	4.85	4.99	1.87	1.92	1.63	2.38	2.83	1.00	3.79	0.0002660
	Mpz	0.14	0.45	0.90	1.43	1.45	0.67	1.09	1.18	1.18	1.19	1.17	1.16	5.35	7.50	7.21	16.34	5.81	7.78	6.05	5.50	5.86	7.21	6.54	7.69	1.00	7.40	0.0000103
	Npy5r	n.d.	0.02	1.68	0.60	2.67	n.d.	0.09	1.29	0.58	1.34	0.89	0.83	15.62	20.27	9.39	17.23	13.40	13.77	15.01	5.05	11.83	4.42	6.39	12.71	1.00	12.09	0.0000068
	Ednrb	0.70	0.73	1.35	1.43	1.19	1.03	1.00	0.64	0.86	1.16	0.85	1.06	3.30	5.67	3.27	3.86	3.06	4.45	2.60	2.24	2.27	2.63	3.43	3.17	1.00	3.33	0.0000029
	Dcn	0.65	0.64	0.64	0.95	1.03	0.97	2.12	1.13	1.22	0.69	1.28	0.68	3.17	3.03	3.05	3.31	2.41	3.73	6.70	2.91	2.79	2.75	3.33	3.36	1.00	3.38	0.0000060
B	Pde1b	1.65	1.41	0.99	1.41	1.17	0.98	0.88	0.78	0.70	0.68	0.60	0.74	0.06	0.07	0.03	0.05	0.04	0.05	0.03	0.03	0.02	0.02	0.03	0.04	1.00	0.04	0.0000009
	Lrtm1	1.97	1.92	0.95	1.33	1.32	0.61	0.93	0.73	0.62	0.56	0.48	0.56	0.46	0.45	0.20	0.22	0.14	0.12	0.11	0.08	0.08	0.07	0.08	0.10	1.00	0.17	0.0001747
	Pcdh20	1.69	1.87	0.92	1.42	0.74	0.80	1.32	0.58	0.61	0.51	0.87	0.66	0.14	0.09	0.07	0.06	0.07	0.09	0.05	0.05	0.03	0.06	0.08	0.10	1.00	0.07	0.0000225
	Mbp	0.56	0.68	0.97	1.50	1.19	0.73	1.12	1.07	0.92	1.10	0.97	1.19	0.03	0.04	0.02	0.03	0.02	0.02	0.03	0.02	0.02	0.02	0.02	0.03	1.00	0.03	0.0000000
	Tmem158	1.42	2.18	1.00	1.48	1.27	0.69	0.87	0.83	0.68	0.53	0.54	0.49	0.27	0.30	0.19	0.20	0.15	0.12	0.09	0.11	0.09	0.08	0.10	0.13	1.00	0.15	0.0001108
	Stc2	1.94	1.73	0.90	1.30	1.21	0.78	0.93	0.84	0.69	0.55	0.52	0.61	0.29	0.28	0.13	0.16	0.11	0.12	0.10	0.07	0.06	0.05	0.07	0.09	1.00	0.13	0.0000344
	Klhl30	1.12	1.16	1.03	1.51	1.10	1.03	1.25	0.85	0.87	0.89	0.70	0.49	0.18	0.19	0.14	0.19	0.17	0.15	0.13	0.11	0.08	0.07	0.09	0.13	1.00	0.14	0.0000002
	Ppp1r1a	1.21	1.16	0.80	1.28	1.20	1.00	1.27	0.94	0.88	0.78	0.63	0.86	0.16	0.12	0.11	0.08	0.08	0.09	0.08	0.10	0.04	0.04	0.03	0.08	1.00	0.08	0.0000000
	Atp1b1	2.31	1.67	0.88	1.40	1.27	0.85	0.92	0.67	0.56	0.46	0.44	0.57	0.25	0.20	0.16	0.15	0.14	0.13	0.12	0.07	0.08	0.07	0.08	0.09	1.00	0.13	0.0002318
	Rcan2	2.59	1.66	0.78	1.06	1.10	0.83	0.83	0.74	0.56	0.65	0.54	0.66	0.13	0.12	0.09	0.09	0.09	0.07	0.05	0.04	0.04	0.04	0.06	0.06	1.00	0.07	0.0001965
	Rgs8	1.09	0.97	0.96	1.27	1.19	1.00	0.98	0.95	0.81	0.88	0.91	0.97	0.03	0.02	0.02	0.02	0.02	0.02	0.02	0.01	0.01	0.01	0.02	0.02	1.00	0.02	0.0000000
	Nme7	1.33	1.27	1.01	1.28	1.04	0.95	1.06	0.91	0.86	0.71	0.69	0.89	1.46	1.36	0.94	1.19	0.93	0.98	0.87	0.68	0.65	0.65	0.72	1.20	1.00	0.97	0.7640704
	Mgp	1.39	1.48	0.67	1.21	0.90	0.76	1.36	0.96	1.01	0.66	0.93	0.66	0.19	0.28	0.16	0.19	0.17	0.17	0.26	0.15	0.15	0.12	0.21	0.18	1.00	0.19	0.0000010
	Wnt16	1.10	0.93	0.94	1.20	1.18	1.07	1.21	1.09	0.75	0.86	0.75	0.92	0.50	0.42	0.48	0.50	0.48	0.45	0.37	0.29	0.27	0.27	0.33	0.43	1.00	0.40	0.0000000
	Klk4	2.28	1.64	0.83	1.12	1.13	0.79	0.91	0.94	0.69	0.54	0.48	0.65	0.63	0.52	0.39	0.44	0.37	0.37	0.25	0.19	0.16	0.17	0.20	0.29	1.00	0.33	0.0007995
	Olig1	1.70	1.61	0.92	1.26	0.97	1.00	1.36	0.83	0.75	0.44	0.76	0.40	1.09	1.15	0.52	0.50	0.35	0.49	0.48	0.18	0.18	0.12	0.17	0.27	1.00	0.46	0.0021723
	Pdgfb	0.61	0.63	0.98	1.29	1.05	1.00	1.36	0.93	0.97	1.03	1.02	1.14	0.17	0.17	0.17	0.40	0.22	0.34	0.33	0.14	0.13	0.14	0.18	0.26	1.00	0.22	0.0000000
	Acan	1.16	1.01	0.93	1.28	1.24	0.95	0.95	0.79	0.69	1.35	0.74	0.92	0.04	0.04	0.05	0.05	0.05	0.05	0.04	0.02	0.03	0.03	0.04	0.04	1.00	0.04	0.0000000
	Fhl1	0.43	0.85	0.85	1.09	1.06	0.83	1.46	0.95	1.10	1.08	1.08	1.24	0.22	0.17	0.29	0.27	0.20	0.40	0.34	0.28	0.33	0.28	0.37	0.38	1.00	0.29	0.0000005
	Cspg4	0.68	0.58	0.78	1.20	0.97	0.90	1.26	1.21	1.16	1.02	1.07	1.16	0.12	0.11	0.13	0.18	0.13	0.22	0.22	0.18	0.18	0.20	0.20	0.22	1.00	0.17	0.0000000
	Cav1	0.53	0.51	0.76	1.15	1.05	1.13	1.49	1.07	0.99	0.95	1.23	1.15	0.18	0.20	0.29	0.31	0.26	0.47	0.47	0.42	0.36	0.58	0.47	0.72	1.00	0.39	0.0000050

Supplementary Table 4: GO-annotation analysis of differentially expressed genes due to forskolin. Raw data of the Figure 3. In forskolin-induced (A) and forskolin-reduced transcripts (B), a number of genes were associated with GO-annotations for extracellular matrix, extracellular region and plasma membrane. Several forskolin-reduced genes were associated with intracellular signaling, such as the MAPKKK cascade (B).

A Induced gene transcripts

Molecular Function

Category	Identification	Term	Count	%	p-value
GOTERM_MF_FAT	GO:0005509~	calcium ion binding	21	8.50	6.26E-03
GALNT2, SVEP1, NCALD, NID1, MMP15, DLK1, SLIT2, CDH5, SLIT3, THBD, GNPTAB, SLC24A3, CACNA1H, RELN, EGFL8, VCAN, SPON2, ENTPD2, PROS1, DST, AOC3					
GOTERM_MF_FAT	GO:0005125~	cytokine activity	14	5.67	4.13E-07
BMP4, CXCL1, IL6, GDF1, CX3CL1, IL33, KITL, CXCL12, CCL7, IL11, CCL11, CXCL14, PPBP, CLCF1					
GOTERM_MF_FAT	GO:0008009~	chemokine activity	7	2.83	9.46E-06
CCL11, CXCL1, CXCL14, PPBP, CX3CL1, CXCL12, CCL7					
GOTERM_MF_FAT	GO:0008092~	cytoskeletal protein binding	15	6.07	8.96E-04
CLMN, BCL2L11, FARP2, CORO2B, FMN2, TRIM2, SYN1, TMOD2, MFAP2, EPB4.1L4B, CLASP1, LOC100044177, ADD3, DST, SYNPO					
GOTERM_MF_FAT	GO:0003779~	actin binding	9	3.64	3.25E-02
CORO2B, FMN2, SYN1, CLMN, TMOD2, LOC100044177, DST, ADD3, SYNPO					
GOTERM_MF_FAT	GO:0050840~	extracellular matrix binding	3	1.21	4.27E-02
NID1, DCN, SHH					

Cellular Component

Category		Term	Count	%	p-value
GOTERM_CC_FAT	GO:0031012~	extracellular matrix	21	8.50	7.29E-09
BMP4, MATN2, FMOD, CTHRC1, LUM, ADAMTS15, NID1, MMP15, DCN, PRELP, LAMA1, COL14A1, CRISPLD2, PTN, TGFBR3, RELN, MFAP2, VCAN, SPON2, ENTPD2, PRSS12					
GOTERM_CC_FAT	GO:0005576~	extracellular region	64	25.91	1.97E-15
CTHRC1, AEBP1, GDF1, FGF7, CRHBP, CXCL12, SHH, IL11, OLFML3, CLCF1, SOSTDC1, LGI4, SEMA3B, SPON2, PRSS35, MATN2, MMP15, SLIT2, SLIT3, PRELP, THBD, PPBP, PLA2G7, STC1, RELN, VCAN, MFAP2, EGFL8, CTSB, PROS1, HSD17B11, CXCL1, GALNT2, DHH, FMOD, LUM, ADAMTS15, CRLF1, CX3CL1, DCN, IL33, CCL7, CRISPLD2, C1QTNF1, PTN, ENTPD2, PRSS12, PRL2C2, BMP4, IL6, SVEP1, NID1, KITL, SOD3, CCL11, LAMA1, COL14A1, CXCL14, SFRP1, FJX1, LIPH, TGFBR3, PLAU, IGFBP5					
GOTERM_CC_FAT	GO:0005886~	plasma membrane	51	20.65	2.76E-02
SLC16A11, FGFRL1, GJA1, DLK1, SHH, EDNRB, UGT1A6B, SLC24A3, UGT1A6A, GNG2, ROBO2, LOC100044177, CEACAM2, RAP2A, MMP15, PTPRU, PTPRN, NCAM1, UGT1A10, THBD, SGSM3, CTSB, KCNH2, DST, ADD3, PMEPA1, AOC3, DHH, ITGB4, KCNA6, CX3CL1, CDH5, ALCAM, IGSF11, SYN1, ITGB8, SYNPO, TMEM45A, LOC100047606, IL6, SMAD3, NPY1R, KITL, NPY5R, CAMK2N1, FZD6, GPR153, SLC17A8, KCNN4, ABCC4, TGFBR3, CACNA1H, TNK2					
GOTERM_CC_FAT	GO:0005615~	extracellular space	28	11.34	5.93E-10
CXCL1, DHH, GDF1, CRHBP, IL33, CX3CL1, CXCL12, SHH, CCL7, IL11, CLCF1, SOSTDC1, C1QTNF1, PRL2C2, BMP4, IL6, KITL, SLIT2, SOD3, SLIT3, CCL11, THBD, CXCL14, PLA2G7, FJX1, RELN, STC1, IGFBP5					

Biological Process

Category		Term	Count	%	p-value
GOTERM_BP_FAT	GO:0010628~	positive regulation of gene expression	12	4.86	7.48E-02
BMP4, HDAC4, IL6, CEBPB, KLF13, GLIS1, BCL3, SMAD3, RORB, SHH, IL11, PRL2C2					
GOTERM_BP_FAT	GO:0007155~	cell adhesion	22	8.91	2.82E-05
AEBP1, SVEP1, ITGB4, NID1, CX3CL1, PTPRU, KITL, LOC100045228, CDH5, BCL2L11, ALCAM, NCAM1, LAMA1, IGSF11, COL14A1, ITGB8, RELN, ROBO2, VCAN, SPON2, DST, AOC3					
GOTERM_BP_FAT	GO:0016477~	cell migration	10	4.05	5.42E-03
EDNRB, GJA1, RELN, CX3CL1, NR2F2, CXCL12, KITL, PLAU, SHH, DCLK1					
GOTERM_BP_FAT	GO:0042127~	regulation of cell proliferation	13	5.26	6.71E-02
BMP4, IL6, FGF7, FGFRL1, SMAD3, KITL, SHH, CDH5, IL11, HDAC4, TGFBR3, PLAU, PRL2C2					
GOTERM_BP_FAT	GO:0007267~	cell-cell signaling	13	5.26	6.81E-04
BMP4, DHH, IL6, EGR3, FGF7, GJA1, FZD2, LOC100045228, SHH, NPY5R, SYN1, TMOD2, PPP3CA					

B Reduced gene transcripts

Molecular Function

Category		Term	Count	%	p-value
GOTERM_MF_FAT	GO:0005509~	calcium ion binding	21	9.17	4.67E-03
PCDH20, ITGA1, ANXA1, MGP, PADI2, MMP17, LPCAT2, PCDH17, CALB2, MYL9, GALNTL4, ITGA5, GSN, LOC638935, CDH19, VSNL1, TGM2, THBS1, CDH10, SNTA1, EHD4, DTNA					

Cellular Component

Category		Term	Count	%	p-value
GOTERM_CC_FAT	GO:0031012~	extracellular matrix	18	7.86	3.98E-07
WNT16, WNT10B, PTPRZ1, TNC, ADAMTSL5, COL15A1, CCDC80, COL28A1, MMP17, COL5A2, COL4A5, THSD4, ACAN, TGM2, ADAMTS1, AGRN, ADAMTS12, COL8A1					
GOTERM_CC_FAT	GO:0005576~	extracellular region	39	17.03	2.33E-04
WNT16, PDGFB, PDGFA, PGF, TNC, ADAMTSL5, CD109, COL28A1, GDNF, FAM132B, C1QTNF3, GSN, TGM2, ACAN, LOXL4, ADAMTS12, AGRN, HTRA3, COL8A1, THBS1, KLK8, WNT10B, STC2, PTPRZ1, COL15A1, CCDC80, IGF1, MMP17, MGP, ENDOD1, COL5A2, COL4A5, 1190002N15RIK, SRPX2, THSD4, NPPB, ADAMTS1, PRSS23, IGFBP3					
GOTERM_CC_FAT	GO:0005604~	basement membrane	9	3.93	3.82E-06
TNC, ACAN, COL15A1, CCDC80, COL28A1, ADAMTS1, AGRN, COL8A1, COL4A5					
GOTERM_CC_FAT	GO:0005886~	plasma membrane	54	23.58	2.29E-03
LOC100047619, LYPD1, ATP1B1, GABRB3, CSPG4, LGR6, KCNJ12, CALB2, MCF2L, MBP, DIRAS1, DYSF, DES, SV2B, RHOC, GPR176, INPPL1, CRYAB, RXFP3, MDGA2, MMP17, RFTN1, TNS3, CD80, GPBAR1, VAMP5, HSPB1, GAP43, ACVR1, CAV1, LIMS2, PCDH20, CD109, ITGB5, PTK2, P2RY1, TGM2, PPAP2A, DTNA, CNKSR2, IRGM1, FLT1, ANXA1, ITGA1, ATP1A2, PCDH17, ABCB4, P2RX5, ITGA5, RGS3, CDH19, CD200, CDH10, SNTA1					
GOTERM_CC_FAT	GO:0008305~	integrin complex	3	1.31	4.90E-02
ITGA5, ITGA1, ITGB5					

Biological Process

Category		Term	Count	%	p-value
GOTERM_BP_FAT	GO:0010628~	positive regulation of gene expression	11	4.80	8.37E-02
MEF2C, WNT10B, ETS1, MEOX1, IRF6, JUN, FOXO1, IGF1, CREB5, AGRN, HMGA1					
GOTERM_BP_FAT	GO:0007242~	intracellular signaling cascade	22	9.61	4.75E-03
CNKSR2, 5430435G22RIK, CAV1, TNIK, RXFP3, STMN4, CSPG4, SPSB4, PEA15A, RASL12, RCAN2, MCF2L, TNS3, DIRAS1, NUPR1, DOK5, GAB1, TGM2, GUCY1A3, MAPK9, RHOC, GADD45B					
GOTERM_BP_FAT	GO:0000165~	MAPKKK cascade	6	2.62	1.39E-02
CAV1, DOK5, GAB1, CSPG4, MAPK9, GADD45B					
GOTERM_BP_FAT	GO:0007155~	cell adhesion	14	6.11	2.30E-02
INPPL1, PCDH20, TNC, ITGA1, COL15A1, COL28A1, ITGB5, PCDH17, ITGA5, CDH19, ACAN, THBS1, COL8A1, CDH10					
GOTERM_BP_FAT	GO:0016477~	cell migration	11	4.80	8.43E-04
TNS3, PTK2, FLT1, PDGFB, PLXNA2, CSPG4, ITGA1, PRKG1, APBB2, GDNF, ACVR1					
GOTERM_BP_FAT	GO:0042127~	regulation of cell proliferation	16	6.99	2.94E-03
CAV1, PDGFB, PDGFA, INPPL1, ANXA1, IGF1, FOXO1, PMAIP1, TNS3, CDKN1A, NUPR1, CD80, IRF6, JUN, TGM2, IGFBP3					
GOTERM_BP_FAT	GO:0007267~	cell-cell signaling	10	4.37	1.04E-02
KLK8, PTK2, WNT10B, GABRB3, PDGFA, TNC, SV2B, AGRN, ATP1A2, GDNF					
